# Genetic Polymorphisms of *IFNG*, *IFNGR1*, and Androgen Receptor and Chronic Prostatitis/Chronic Pelvic Pain Syndrome in a Chinese Han Population

**DOI:** 10.1155/2021/2898336

**Published:** 2021-10-04

**Authors:** Lei Chen, Junyi Chen, Fan Mo, Zichen Bian, Chen Jin, Xianguo Chen, Chaozhao Liang

**Affiliations:** ^1^Department of Urology, The First Affiliated Hospital of Anhui Medical University, Hefei, 230022 Anhui, China; ^2^Institute of Urology, Anhui Medical University, Hefei, 230022 Anhui, China; ^3^Anhui Province Key Laboratory of Genitourinary Diseases, Anhui Medical University, Hefei, 230022 Anhui, China

## Abstract

**Background:**

Chronic prostatitis/chronic pelvic pain syndrome (CP/CPPS) refers to a common disorder with unclear etiology and unsatisfactory treatment, which reduces the male's quality of life.

**Objective:**

To examine the effects of genetic polymorphisms of *IFNG*, *IFNGR1*, and androgen receptor (*AR*) on CP/CPPS.

**Methods:**

The single nucleotide polymorphisms (SNPs) of *IFNG*, *IFNGR1*, and *AR* were genotyped with the improved multiplex ligation detection reaction. The GTEx, RegulomeDB, HaploReg, and 3DSNP databases were adopted to predict the regulatory functions of the genotyped SNPs. The correlation between SNPs and CP/CPPS was analyzed with the *χ*^2^ test, logistic regression, and two genetic models (codominant and log-additive models). The nomogram was built to predict the risk of CP/CPPS occurrence.

**Results:**

On the whole, 130 CP/CPPS patients and 125 healthy controls were recruited in the study, and 18 SNPs of *IFNG*, *IFNGR1*, and *AR* were genotyped. The results of functional annotation indicated that the 18 genotyped SNPs might have regulatory effects in the whole blood. The rs144488434 was correlated with the elevated CP/CPPS risk (odds ratio (OR): 2.40, 95% confidence interval (CI): 1.12-5.13, *χ*^2^ = 5.37, and *P* = 0.021) by the *χ*^2^ test. In the built genetic models, rs10457655 was correlated with the elevated National Institutes of Health Chronic Prostatitis Symptom Index (NIH-CPSI) scores (codominant model: GA/GG: crude mean difference (MD) = 0.98, 95% CI: -1.71-3.67 and AA/GG: crude MD = 9.10, 95% CI: 0.58-17.62, *P* = 0.10). In subgroup analysis, rs2069718 was correlated with the elevated CP/CPPS risk (log-additive model: crude OR = 2.18, 95% CI: 1.03-4.64, and *P* = 0.034) in patients ≥ 35 years. The nomogram integrating age, rs2069718, rs10457655, and rs144488434 showed good performance to predict the risk of CP/CPPS.

**Conclusions:**

Genetic polymorphisms of *IFNG*, *IFNGR1*, and *AR* might act as the genetic factors for CP/CPPS susceptibility, which deserved further explorations.

## 1. Introduction

According to the National Institutes of Health (NIH) classification, chronic prostatitis/chronic pelvic pain syndrome (CP/CPPS) is defined as category III prostatitis, affecting approximately 8.4% to 25% of males worldwide [1, 2]. The clinical manifestations of CP/CPPS vary significantly, which consist of pain or discomfort in the perineal or pelvic region, sexual dysfunction, and others, thereby affecting males' quality of life (QoL) [3]. As the etiology of CP/CPPS has been extensively investigated, genetic predisposition towards CP/CPPS was proposed. By analyzing genetic polymorphisms of tumor necrosis factor- (TNF-) *α* and interleukin- (IL-) 10, Shoskes et al. reported that CPPS patients had a higher proportion of low IL-10 production genotype, and the symptoms of patients with high IL-10 and low TNF-*α* phenotypes were not mitigated by anti-inflammatory phytotherapy [4]. Additionally, polymorphisms of manganase superoxide dismutase and genes in the X chromosome might be the genetic factors correlated with CP/CPPS development [5, 6]. Hence, genetic polymorphism might contribute to the pathogenesis of CP/CPPS, and a genome-wide association study (GWAS) should be conducted to identify CP/CPPS-related high-risk alleles and genotypes to facilitate its diagnosis and treatment.

Interferon-*γ* (IFN-*γ*, encoded by *IFNG*) and its receptor (encoded by *IFNGR1*) exert vital effects on the inflammatory response against pathogen infection [7]. According to the experimental autoimmune prostatitis (EAP) model [8], the IFN-*γ* level increased in the prostate, and IFN-*γ*-secreting cells specific to the prostate were detected in the spleen, thereby determining the key effects of IFN-*γ* on the induction of autoimmune prostatitis [9]. For CP/CPPS patients, IFN-*γ* levels were upregulated in the seminal plasma, and prostate antigen-specific IFN-*γ*-secreting lymphocytes were identified in the peripheral blood [10]. In our previous studies, the proportion of IFN-*γ*^+^-T-helper 1 (Th1) cells was reported to increase in the periphery blood of CP/CPPS patients [11]. Thus, IFN-*γ* significantly affected the pathogenesis of CP/CPPS. Several single nucleotide polymorphisms (SNPs) of *IFNG* and *IFNGR1* (e.g., rs3799488, rs9376267, and rs9376268) were correlated with the risk of pulmonary tuberculosis and colon and rectal cancer [12–14]. Genetic polymorphisms of AR were associated with the risk of testicular cancer and male infertility [15, 16]. Riley and Krieger highlighted the genes located in the X chromosome (e.g., the androgen receptor (*AR*)) should be studied in depth to examine the relationship between genetic polymorphisms and CP/CPPS susceptibility [6]. However, the study devoted to elucidating the correlation between polymorphisms of *IFNG*, *IFNGR1*, and *AR* and CP/CPPS has been rarely conducted, and the effects of genetic polymorphism in CP/CPPS deserved further exploration to gain insights into CP/CPPS development.

In the present study, the SNPs of *IFNG*, *IFNGR1*, and *AR* and CP/CPPS risk were analyzed, and a nomogram was built to predict the risk of CP/CPPS based on age and the identified SNPs, which exhibited high performance.

## 2. Materials and Methods

### 2.1. Participants and Samples

All patients diagnosed as CP/CPPS originated from the urology clinic of the First Affiliated Hospital of Anhui Medical University between September 2019 and January 2020. The ethnicity and residency-matched healthy subjects came from the healthy check-up center of our hospital. All individuals were Han Chinese and lived in Anhui province. After the history was critically reviewed and the physical examination was performed, CP/CPPS patients were diagnosed by a senior urologist based on the NIH classification [1, 17]. All patients completed the National Institutes of Health Chronic Prostatitis Symptom Index (NIH-CPSI) questionnaire [18]. The inclusion and exclusion criteria are presented below: the inclusion criteria: (i) patients suffering ≥3 months of symptoms in the last 6 months, covering pain or discomfort in the perineal, lower abdominal, or pelvic region, with the NIH − CPSI score ≥ 10 and (ii) normal results of expressed prostatic secretion (EPS) and urine analyses. The exclusion criteria: patients with other urological disorders consisting of acute or chronic bacterial prostatitis, urinary tract infection or urethritis in the last year, interstitial cystitis, urolithiasis, history of urogenital cancer, and prostate surgery history, as well as neurological disorders. All patients recruited provided the written informed consents, and this study was approved by the ethics committee of the First Affiliated Hospital of Anhui Medical University (PJ-2021-08-45).

### 2.2. Genotyping

Genomic DNA was isolated from the 5 ml of collected venous blood and stored at -80°C to be genotyped. On the whole, 18 SNPs (2 SNPs of *IFNG*, 6 SNPs of *IFNGR1*, and 10 SNPs of *AR*) were genotyped with the improved multiplex ligation detection reaction (iMLDR) system by adopting the multiplex polymerase chain reaction- (PCR-) ligase detection method (Shanghai Genesky Biotechnologies Inc., China). The different fluorescent labels (FAM, VIC, NED, and PET) and different extended 3′-end lengths were exploited to distinguish alleles and SNPs, respectively. The double-distilled water-replaced templates and the primer-free samples acted as the two negative controls. Duplicate tests were performed to verify the consistency of the results. The GeneMapper 4.0 software (Applied Biosystems, America) was adopted to process the raw data.

### 2.3. Functional Annotation of the 18 Genotyped SNPs

To assess the effects of genetic variants of *IFNG*, *IFNGR1*, and *AR* in CP/CPPS-related symptoms, the correlation between genotypes and NIH-CPSI scores was analyzed. The GTEx database [19] was adopted to explore the effects of genetic variants on the expressions of their corresponding genes in whole blood. The HaploReg [20], RegulomeDB [21], and 3DSNP [22] were used to examine the potential effects of the 18 genotyped SNPs in our study. The HaploReg v4.1 online tool is aimed at annotating the noncoding genome by predicting the potential target genes, relevant variants in the haplotype block, etc. Moreover, the annotation of the 18 genotyped SNPs in HaploReg was performed by setting the parameters below: East Asian population, *r*^2^ > 0.8, and the 15-state core model source for epigenome analysis. The 3DSNP database was employed to predict the genetically related SNPs by three-dimensional (3D) chromatin looping.

### 2.4. ELISA Assay

The enzyme-linked immunosorbent assay (ELISA) assay kit (cat# E-EL-H0108c; Elabscience Biotechnology) was used to detect the serum IFN-*γ* concentration in CP/CPPS patients and healthy controls at 450 nm, which was performed by complying with the manufacturer's manual.

### 2.5. Statistical Analysis

For continuous variables, data were expressed as mean ± standard deviation (SD). The correlation between allele and genotype frequencies and CP/CPPS was assessed by the *χ*^2^ test and the logistic regression to establish the genetic model with the SPSS (version 22.0, IBM Corp., Armonk, NY, USA) and the SNPStats tool [23]. The additive model performed better in detecting dominant and additive effects in the unknown inheritance pattern of the variant, whereas it remained poor in examining the recessive effects [24, 25]. Lettre et al. found that the codominant model outperformed other models [26]. Hence, we used the additive model and codominant model to examine the correlation between the genotyped SNPs and CP/CPPS. The two genetic models were defined as follows: codominant model: XX versus YX and XX versus YY and log-additive model: for each Y allele increase (wild allele: X and minor allele: Y). The Haploview (version 4.2) was used to perform the Hardy-Weinberg equilibrium (HWE) test and visualize the linkage disequilibrium (LD) among SNPs. The SHEsis software platform was adopted to perform haplotype construction [27], and the lowest frequency threshold was set to 0.03. The nomogram was built with the *rms*, *rmda*, and *pROC* packages in the R 3.6.3 software.

## 3. Results

### 3.1. Basic Information

A total of 130 patients diagnosed with CP/CPPS and 125 healthy controls were recruited in this study. The age was 34.62 ± 9.27 years in the patient group and 37.89 ± 10.79 years in the control group; the NIH-CPSI score was 21.64 ± 6.33 in CP/CPPS patients (Table 1). The information of the 18 SNPs, minor allele frequency (MAF), and HWE test are listed in Supplemental Table 1. Because all subjects in the present study were male, HWE was not assessed in SNPs located in the X chromosome. The primers applied in this study are listed in Supplemental Table 2.

### 3.2. Potential Effects of the 18 Genotyped SNPs

According to Supplemental Figure 1, no significant correlation was identified between genotypes of the 18 genotyped SNPs and NIH-CPSI scores. The results of the GTEx database analysis revealed that rs1861493, rs2069718, rs7749390, rs9376267, and rs9376268 might affect the expression of the corresponding genes (Supplemental Figure 2). According to Supplemental Table 3, the RegulomeDB scores ranged from 2b to 7 of the 18 genotyped SNPs. According to RegulomeDB, the higher score represents a less regulatory function of a variant [21]. For rs10457655, the RegulomeDB score reached 2b, thereby demonstrating its abilities of TF binding, any motif, a DNase Footprint, and a DNase peak. rs3799488, with a score of 3a, had roles of transcription factor (TF) binding, any motif, and a DNase peak. For the rest of SNPs with 4 to 6 scores, only less binding evidence was reported. The HaploReg results revealed that most of the 18 genotyped SNPs were correlated with motif changed and histone markers, and some variants might act as expression quantitative trait loci (eQTL) to regulate the expression of related genes. Additionally, the levels of serum IFN-*γ* were also detected in CP/CPPS patients and healthy controls, and no significant difference in serum IFN-*γ* levels was found between CP/CPPS patients and healthy controls, and genotypes of rs1861493 and rs2069718 were not correlated with the serum IFN-*γ* levels in CP/CPPS patients (Supplemental Figure 3). Taken together, these 18 SNPs were predicted to have potential regulatory functions, while their effects on CP/CPPS warranted further study.

### 3.3. Distributions of Allele and Genotype Frequencies and Genetic Model

According to Table 2, as revealed from the results, rs144488434 was correlated with the elevated CP/CPPS risk (T/C, odds ratio (OR): 2.40, 95% confidence interval (CI): 1.12-5.13, *χ*^2^ = 5.37, and *P* = 0.021), and no significant correlation was reported between the remaining SNPs and CP/CPPS. The codominant and log-additive models were built to examine the correlation among SNPs, CP/CPPS, and NIH-CPSI scores. The Akaike's Information Criterion (AIC) and Bayesian Information Criterion (BIC) values were calculated to determine the appropriate model. As indicated from our results, rs10457655 was correlated with the elevated NIH-CPSI scores in CP/CPPS patients without being adjusted by age (codominant model: GA/GG: crude MD = 0.98, 95% CI: -1.71-3.67 and AA/GG: crude MD = 9.10, 95% CI: 0.58-17.62, *P* = 0.10) (Tables 3 and 4). Accordingly, rs10457655 might be correlated with CP/CPPS.

### 3.4. Subgroup Analysis

Based on the findings above, we conducted a subgroup analysis according to CP/CPPS patients' age. As shown in Supplemental Table 4 and 5, in participants ≥ 35 years (*N*_patient_ = 62 and *N*_control_ = 62), rs2069718 was correlated with the elevated CP/CPPS risk without being adjusted by age (log-additive model: crude OR = 2.18, 95% CI: 1.03-4.64, and *P* = 0.034), while no significant correlation was found in participants < 35 years (*N*_patient_ = 68 and *N*_control_ = 63).

### 3.5. LD and Haplotype Analysis

**Figure 1:**
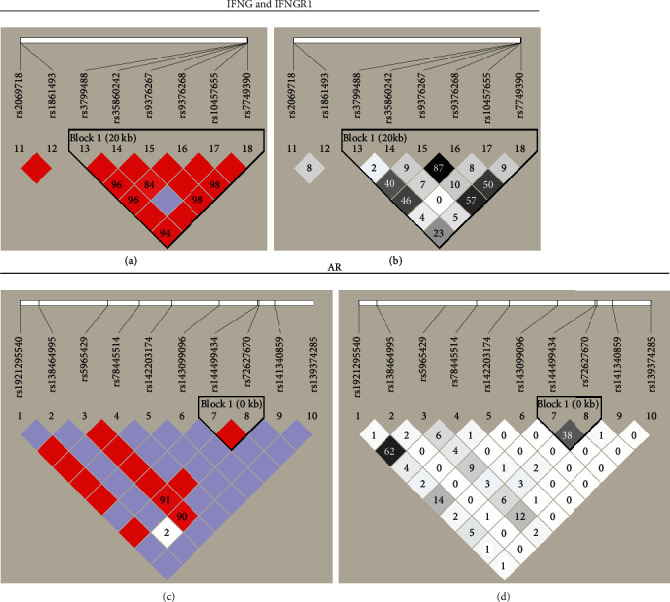
Linkage disequilibrium (LD) plots for single nucleotide polymorphisms. (SNPs) of (a, b) *IFNG* and *IFNGR1* and (c, d) *AR*. (a, c) represent the *D*′ values, and (b, d) represent the *r*^2^ values.

We analyzed the LDs among the 18 genotyped SNPs, and LD was defined as *D*′ ≥ 0.9 and *r*^2^ ≥ 0.6. Strong LDs were found between rs9376267 and rs9376268 in *IFNGR1* (*D*′ = 1.00 and *r*^2^ = 0.873) and between rs192195540 and rs5965429 in *AR* (*D*′ = 1.00 and *r*^2^ = 0.629) (Figures 1(a)–1(d)). There was no correlation between rs9376267-rs9376268 and rs192195540-rs5965429 genotype distribution frequencies in CP/CPPS patients and healthy controls (Supplemental Table 6). Additionally, we predicted that the rs1861493, rs2069718, rs9376267, rs9376268, and rs10457655 might exert regulatory effects through interacting with their genetically related SNPs (LD *r*^2^ > 0.8) in the 3DSNP database (Supplemental Table 7-11).

### 3.6. Predictive Nomogram

**Figure 2:**
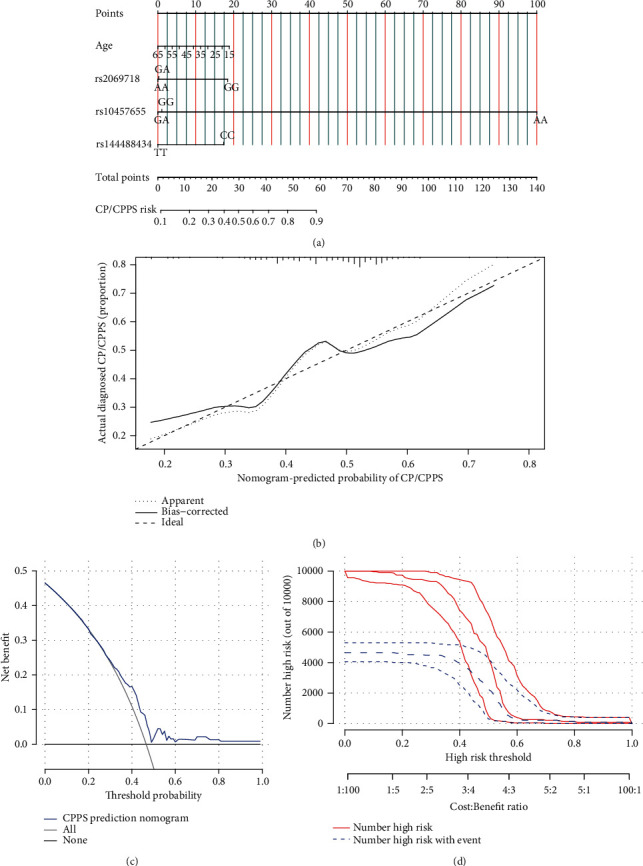
The SNP-based nomogram predicted the risk of CP/CPPS. (a) The nomogram with four variables was built to predict the risk of CP/CPPS. (b) The calibration curves indicated the agreement between the predicted CP/CPPS risk and the actual risk. (c, d) Decision curve and clinical impact curve revealed the net benefit and the nomogram-predicted probability.

To gain insights into the effects of SNPs on CP/CPPS, the SNP-based nomogram was built to predict the risk of CP/CPPS. Since rs2069718, rs10457655, and rs144488434 were identified to be possibly correlated with CP/CPPS, age, rs2069718, rs10457655, and rs144488434 were adopted to build the nomogram (Figure 2(a)). The calibration curve of the nomogram indicated the agreement of our cohort (Figure 2(b)), and the concordance index (C-index) was 0.62 (95% CI: 0.54-0.68), and the area under the curve (AUC) of receiver operating characteristics (ROC) reached 0.62 (95% CI: 0.55-0.69). Based on the decision curve and the clinical impact curve, the clinical usefulness of the nomogram of this study was visualized, i.e., the model showed good net benefit (Figures 2(c) and 2(d)). The internal validation was performed to assess the goodness-of-fit of the built nomogram by splitting our cohort into training and validation cohorts at different ratios, and the C-indexes and AUC values in training and validation cohorts showed adequate internal goodness-of-fit of this study's model (Supplemental Table 12).

## 4. Discussion

In the present study, we investigated the correlation between SNPs and the risk of CP/CPPS, the results of this study were as follows: (1) functional annotation of the 18 genotyped SNPs predicted their regulatory effects in blood, while these SNPs were not correlated with NIH-CPSI score; (2) rs2069718 in *IFNG*, rs10457655 in *IFNGR1*, and rs144488434 in *AR* might be correlated with the increased risk of CP/CPPS; (3) LD was reported between rs9376267 and rs9376268, as well as between rs192195540 and rs5965429; (4) nomogram based on age, rs2069718, rs10457655, and rs144488434 was built to predict the risk of CP/CPPS, which showed good performance. Taken together, we found that genetic polymorphisms of *IFNG*, *IFNGR1*, and *AR* were correlated with CP/CPPS, which might indicate the genetic predisposition to CP/CPPS development and provided novel insight into understanding the etiology of CP/CPPS.

IFN-*γ*, a proinflammatory and immunomodulatory cytokine secreted by natural killer (NK), NKT, and T cells, acts on activation of the innate immune system against pathogen invasion by binding to its receptor [28]. Previous studies have demonstrated that genetic polymorphisms of *IFNG* and *IFNGR1* were correlated with tuberculosis infection, systemic lupus erythematosus, etc. [12, 29, 30]. Based on the important effects of IFN-*γ* in EAP and CP/CPPS [9–11], we determined the effects of genetic polymorphisms of *IFNG* and *IFNGR1* in CP/CPPS. Initially, functional annotation of the 8 genotyped SNPs of *IFNG* and *IFNGR1* was performed by GTEx database, RegulomeDB, and HaploReg tools, which indicated that these 8 SNPs might have regulatory effects in human whole blood. Nevertheless, no significant association among the mentioned variants, NIH-CPSI scores, and serum IFN-*γ* levels was observed, and the serum IFN-*γ* levels were not elevated in CP/CPPS patients. Hence, the 8 genotyped SNPs of *IFNG* and *IFNGR1* might not be correlated with CP/CPPS-related symptoms, and the regulatory effects of the 8 genotyped SNPs in CP/CPPS deserved further study. The results of codominant and log-additive models showed that rs2069718 in *IFNG* was correlated with the elevated CP/CPPS risk, and rs10457655 in *IFNGR1* was correlated with increased NIH-CPSI score. Combined with the autoimmune characteristics of CP/CPPS and the roles of rs2069718 and rs10457655 in inflammatory diseases [30–32], we speculated that rs2069718 and rs10457655 might be involved in the pathogenesis of CP/CPPS through other mechanisms as predicted by the HaploReg tool [20], including protein binding and regulatory motif alterations. We also found that rs1861493, rs2069718, rs9376267, rs9376268, and rs10457655 might interact with their genetically related SNPs by the 3DSNP database, and combined with the significant functions of the SNP-SNP interaction network in the pathogenesis of prostate cancer [33] and inflammatory diseases [34, 35], we speculated that rs2069718 and rs10457655 might function through the SNP-SNP interaction pattern in CP/CPPS.

AR (encode by *AR* gene) can act as a transcription factor and exert its role by regulating androgen-targeted genes [36]. Polymorphisms of *AR* affected hormone level, sperm parameter, and the growth of the prostate [37, 38]. In function annotation of the 10 genotyped SNPs of *AR*, these SNPs were predicted to exhibit the regulatory potential by RegumeDB and HaploReg. Reciprocally, these SNPs did not regulate blood *AR* expression in the GTEx database. Additionally, the mentioned variants in *AR* were found to be not associated with CP/CPPS scores. Thus, the variants of *AR* might not regulate the phenotype of CP/CPPS directly, but through indirect mechanisms. In this study, rs144488434 in *AR* was found to be correlated with the elevated CP/CPPS risk, which might be involved in the pathogenesis of CP/CPPS, and further studies should be conducted to examine the underlying mechanisms. Based on the significant findings of rs2069718, rs10457655, and rs144488434 in CP/CPPS, a nomogram was built, integrating age, and genotypes of rs2069718, rs10457655, and rs144488434 to predict the risk of CP/CPPS, which might apply the findings of the study to clinical practice and assisted clinicians to more effectively identify CP/CPPS patients.

As the common urological disorders in males, approximately 8.4% of subjects had prostatitis-like symptoms in Chinese in our previous study [39]. The risk factors of CP/CPPS have been investigated increasingly (e.g., age, physical inactivity, stress, smoking, and alcohol consumption) [40, 41]. Zhang et al. [42] reported that CP/CPPS is a very problem in Chinese males, with a lifetime prevalence of 25.3%, and some patients might have a good prognosis without treatment, whereas some might require symptom-orientated therapies to facilitate the prognosis. Though therapeutic approaches for CP/CPPS (e.g., anti-inflammatory, antimicrobial agents, and *α*-blockers) had been applied to clinical practice, monotherapy could not effectively mitigate the symptoms, and combination therapy was recommended [43]. The CP/CPPS patients responded differently to the treatments, and the underlying mechanisms remain unclear. The direct (e.g., medications and office visits) and indirect costs (e.g., wage loss) to treat CP/CPPS were substantial to patients, and the unsatisfactory therapeutic efficacy and characteristic of recurrence imposed the heavy burden on patients and society [44, 45]. A cost-efficient treatment to CP/CPPS should be urgently developed to maximize the therapeutic efficacy, and thus, it could reduce the cost [45]. Given the relationship between therapeutic efficacies and IL-10 and TNF-*α* phenotypes, Shoskes et al. identified a subgroup of CP/CPPS patients with high IL-10 and low TNF-*α* genotypes responding poorly to anti-inflammatory phytotherapy [4]. For this reason, patients with different genotypes achieved different therapeutic efficacies. Given the findings of genetic predisposition to CP/CPPS, more studies are aimed at examining the effects of genetic factors on the diagnosis and personalized therapy of CP/CPPS should be conducted [5, 6].

The strengths of this study were that we explored the effects of genetic factors on CP/CPPS, which might provide a novel clue for the diagnosis and treatment of CP/CPPS. Moreover, the functions of the 18 SNPs of *IFNG*, *IFNGR1*, and *AR* were annotated by different databases, and their correlation with the symptoms of CP/CPPS was also analyzed. The nomogram predicting the risk of CP/CPPS was built by integrating age and the identified SNPs, and internal validation showed the adequate goodness-of-fit of the model, which might contribute to the diagnosis of CP/CPPS. The limitations of this study were that only 18 SNPs were genotyped, and other significant SNPs of *IFNG* (e.g., rs2069705 and rs1861494), *IFNGR1* (e.g., rs1327475, rs1327474, and rs2234711), and *AR* (e.g., CAG, GGC, and GGN repeats and G1733A) were not genotyped. Moreover, the sample size was relatively small for a genetic association study, especially in the subgroup analysis based on participants' age. The subjects in this study were from a single hospital in China, and external validation was not applicable, thereby restricting the wide use of the results. Moreover, a multicenter study should be conducted to further identify CP/CPPS-related SNPs. In the future, the effects of genetic epidemiology in CP/CPPS should be highlighted. Environmental factors (e.g., the low altitude, higher education level, and more time in the sunlight) were correlated with reduction of prostatitis-like symptoms [46, 47], while cold exposure facilitated the initiation of CP/CPPS [48]. Thus, the application of genetic epidemiology to CP/CPPS might contribute to determining the genetic susceptibility and genetic model of CP/CPPS.

## 5. Conclusions

Three SNPs were identified (i.e., rs2069718 of *IFNG*, rs10457655 of *IFNGR1*, and rs144488434 of *AR)* to be potentially correlated with CP/CPPS. Genetic polymorphisms of *IFNG*, *IFNGR1*, and *AR* might function as the genetic factors for CP/CPPS susceptibility, and the underlying mechanisms should be further explored.

## Figures and Tables

**Table 1:** Basic information of participants in this study.

	Healthy control	Patients with CP/CPPS
No. of subjects	125 (49.02%)	130 (50.98%)
Age (years)	37.89 ± 10.79	34.62 ± 9.27
NIH-CPSI score	NA	21.64 ± 6.33^a^ 21.00 (17, 24.75)^b^

Data are presented as *n* (%) or mean ± standard deviation (SD)^a^ or median (q1, q3)^b^. CP/CPPS: chronic prostatitis/chronic pelvic pain syndrome; NA: not applicable; NIH-CPSI: National Institutes of Health Chronic Prostatitis Symptom Index.

**Table 2:** The association of allele and genotype frequencies and CP/CPPS.

Gene	SNP	Allele/genotype	Cases	Control	OR (95% CI)	*χ* ^2^	*P*
			*n* (%)	*n* (%)			
*IFNG*	rs2069718	A	204 (86.44)	224 (89.60)	0.74 (0.43, 1.28)	1.153	0.283
		G	32 (13.56)	26 (10.40)			
		AA	89 (75.42)	100 (80.00)		1.520	0.468
		GA	26 (22.03)	24 (19.20)			
		GG	3 (2.55)	1 (0.80)			
	rs1861493	A	146 (61.86)	154 (61.60)	1.01 (0.70, 1.46)	0.004	0.952
		G	90 (38.14)	96 (38.40)			
		AA	46 (38.98)	49 (39.20)		0.035	0.983
		GA	54 (45.76)	56 (44.80)			
		GG	18 (15.26)	20 (16.00)			

*IFNGR1*	rs10457655	G	207 (87.71)	224 (89.60)	0.83 (0.47, 1.45)	0.431	0.511
		A	29 (12.29)	26 (10.40)			
		GG	91 (77.12)	99 (79.20)		2.16	0.340
		GA	25 (21.19)	26 (20.80)			
		AA	2 (1.69)	0 (0.00)			
	rs35860242	C	216 (91.53)	237 (94.80)	0.59 (0.29, 1.22)	2.057	0.152
		T	20 (8.47)	13 (5.20)			
		CC	101 (85.59)	112 (89.60)		3.41	0.182
		CT	14 (11.87)	13 (10.40)			
		TT	3 (2.54)	0 (0.00)			
	rs3799488	T	178 (75.42)	185 (74.00)	1.08 (0.72, 1.62)	0.130	0.718
		C	58 (24.58)	65 (26.00)			
		TT	67 (56.78)	72 (57.60)		1.40	0.496
		CT	44 (37.29)	41 (32.80)			
		CC	7 (5.93)	12 (9.60)			
	rs7749390	G	135 (57.20)	141 (56.40)	1.03 (0.72, 1.48)	0.032	0.858
		A	101 (42.80)	109 (43.60)			
		GG	39 (33.05)	38 (30.40)		0.336	0.845
		GA	57 (48.31)	65 (52.00)			
		AA	22 (18.64)	22 (17.60)			
	rs9376267	C	133 (56.84)	138 (55.20)	1.07 (0.75, 1.53)	0.132	0.717
		T	101 (43.16)	112 (44.80)			
		CC	35 (29.91)	38 (30.40)		0.686	0.710
		CT	63 (53.85)	62 (49.60)			
		TT	19 (16.24)	25 (20.00)			
	rs9376268	G	139 (59.40)	148 (58.73)	1.01 (0.70, 1.45)	0.002	0.964
		A	95 (40.60)	102 (40.80)			
		GG	39 (33.33)	43 (34.12)		0.182	0.913
		GA	61 (52.14)	62 (49.21)			
		AA	17 (14.53)	20 (16.00)			

*AR*	rs138464995	C	204 (94)	238 (95)	0.86 (0.38, 1.95)	0.135	0.713
		T	12 (6)	12 (5)			
		CC	102 (94)	119 (95)	0.86 (0.27, 2.74)	0.068	0.795
		TT	6 (6)	6 (5)			
	rs142203174	A	212 (90)	230 (92)	0.77 (0.41, 1.43)	0.694	0.405
		C	24 (10)	20 (8)			
		AA	106 (90)	115 (92)	0.77 (0.32,1.85)	0.347	0.556
		CC	12 (10)	10 (8)			
	rs192195540	G	190 (81)	192 (77)	1.30 (0.84, 2.03)	1.41	0.236
		C	44 (19)	58 (23)			
		GG	95 (81)	96 (77)	1.30 (0.70, 2.43)	0.702	0.402
		CC	22 (19)	29 (23)			
	rs139374285	C	226 (96)	238 (95)	1.14 (0.48, 2.69)	0.089	0.766
		T	10 (4)	12 (5)			
		CC	113 (96)	119 (95)	1.14 (0.34, 3.84)	0.044	0.833
		TT	5 (4)	6 (5)			
	rs72627670	C	202 (86)	202 (81)	1.50 (0.92, 2.44)	2.67	0.102
		T	32 (14)	48 (19)			
		CC	101 (86)	101 (81)	1.50 (0.75, 2.99)	1.34	0.248
		TT	16 (14)	24 (19)			
	rs144488434	C	226 (96)	226 (90)	2.40 (1.12, 5.13)	5.37	0.021
		T	10 (4)	24 (10)			
		CC	113 (96)	113 (90)	2.40 (0.82, 7.04)	2.68	0.101
		TT	5 (4)	12 (10)			
	rs143099096	C	228 (97)	240 (96)	1.19 (0.46, 3.06)	0.127	0.722
		A	8 (3)	10 (4)			
		CC	114 (97)	120 (96)	1.19 (0.31, 4.53)	0.000	1.000
		AA	4 (3)	5 (4)			
	rs78445514	T	198 (84)	222 (89)	0.66 (0.39, 1.11)	2.49	0.115
		C	38 (16)	28 (11)			
		TT	99 (84)	111 (89)	0.66 (0.31, 1.38)	1.24	0.265
		CC	19 (16)	14 (11)			
	rs141340859	T	216 (92)	238 (95)	0.61 (0.29, 1.29)	1.74	0.187
		C	18 (8)	12 (5)			
		TT	108 (92)	119 (95)	0.61 (0.21, 1.76)	0.870	0.351
		CC	9 (8)	6 (5)			
	rs5965429	A	168 (73)	166 (67)	1.34 (0.90, 1.98)	2.12	0.146
		G	62 (27)	82 (33)			
		AA	84 (73)	83 (67)	1.34 (0.77, 2.34)	1.06	0.304
		GG	31 (27)	41 (33)			

CP/CPPS: chronic prostatitis/chronic pelvic pain syndrome; OR: odds ratio; SNPs: single nucleotide polymorphisms.

**Table 3:** Genetic models of SNPs and CP/CPPS by using logistic regression.

Gene/SNP	Model	Genotype	Crude OR (95% CI)	*P*	AIC	BIC	Adjusted OR (95% CI)^a^	*P*	AIC	BIC
*IFNG*										
rs2069718	Codominant model	GA/AA	1.22 (0.65-2.27)	0.46	341.1	351.6	1.17 (0.62-2.19)	0.37	337.5	351.5
		GG/AA	3.37 (0.34-32.99)				4.33 (0.43-43.20)			
	Log-additive model	-	1.34 (0.78, 2.32)	0.29	339.5	346.5	1.35 (0.78, 2.33)	0.29	336.4	346.9
rs1861493	Codominant model	GA/AA	1.03 (0.59-1.78)	0.98	342.6	353.1	0.99 (0.57-1.72)	1	339.5	353.5
		GG/AA	0.96 (0.45-2.04)				1.03 (0.48-2.20)			
	Log-additive model	-	0.99 (0.69, 1.42)	0.95	340.7	347.7	1.01 (0.70, 1.45)	0.97	337.5	348
*IFNGR1*										
rs10457655	Codominant model	GA/GG	1.05 (0.56-1.94)	0.23	339.7	350.2	1.04 (0.55-1.93)	0.34	337.4	351.4
		AA/GG	NA				NA			
	Log-additive model	-	1.22 (0.68, 2.17)	0.50	340.2	347.2	1.17 (0.65, 2.10)	0.60	337.3	347.7
rs35860242	Codominant model	CT/CC	1.19 (0.54-2.66)	0.1	338.1	348.6	1.14 (0.51-2.55)	0.16	335.9	349.9
		TT/CC	NA				NA			
	Log-additive model	-	1.60 (0.80, 3.19)	0.17	338.8	345.8	1.48 (0.74, 2.99)	0.26	336.3	346.8
rs3799488	Codominant model	CT/TT	1.15 (0.67-1.98)	0.49	341.3	351.7	1.26 (0.73-2.19)	0.41	337.8	351.7
		CC/TT	0.63 (0.23-1.69)				0.65 (0.24-1.77)			
	Log-additive model	-	0.93 (0.63, 1.38)	0.73	340.5	347.5	0.98 (0.65, 1.46)	0.90	337.5	348
rs7749390	Codominant model	GA/GG	0.85 (0.48-1.51)	0.85	342.3	352.8	0.89 (0.50-1.58)	0.91	339.4	353.3
		AA/GG	0.97 (0.46-2.04)				0.97 (0.46-2.05)			
	Log-additive model	-	0.97 (0.67, 1.39)	0.86	340.6	347.6	0.97 (0.67, 1.40)	0.87	337.5	348
rs9376267	Codominant model	CT/CC	1.10 (0.62-1.97)	0.71	340.5	351	1.15 (0.64-2.06)	0.65	337.5	351.5
		TT/CC	0.83 (0.39-1.75)				0.83 (0.39-1.78)			
	Log-additive model	-	0.93 (0.65, 1.35)	0.71	339.1	346.1	0.94 (0.65, 1.36)	0.74	336.3	346.7
rs9376268	Codominant model	GA/GG	1.08 (0.62-1.90)	0.91	341	351.5	1.08 (0.61-1.90)	0.89	338.1	352.1
		AA/GG	0.94 (0.43-2.04)				0.90 (0.41-1.99)			
	Log-additive model	-	0.99 (0.68, 1.44)	0.96	339.2	346.2	0.98 (0.67, 1.42)	0.90	336.4	346.8
*AR*										
rs138464995		TT/CC	1.17 (0.36-3.73)	0.8	325.7	332.6	1.07 (0.33-3.45)	0.91	322.6	333
rs142203174		CC/AA	1.30 (0.54-3.14)	0.56	340.3	347.3	1.38 (0.57-3.38)	0.48	337	347.5
rs192195540		CC/GG	0.77 (0.41-1.43)	0.4	338.5	345.5	0.69 (0.37-1.30)	0.25	335.1	345.5
rs139374285		TT/CC	0.88 (0.26-2.96)	0.83	340.6	347.6	0.90 (0.26-3.08)	0.87	337.5	348
rs72627670		TT/CC	0.67 (0.33-1.33)	0.25	337.9	344.9	0.70 (0.35-1.42)	0.32	335.4	345.9
rs144488434		TT/CC	0.42 (0.14-1.22)	0.096	337.9	344.9	0.39 (0.13-1.16)	0.076	334.4	344.9
rs143099096		AA/CC	0.84 (0.22-3.21)	0.8	340.8	347.6	0.68 (0.17-2.62)	0.57	337.2	347.7
rs78445514		CC/TT	1.52 (0.72-3.19)	0.26	339.4	346.4	1.52 (0.72-3.22)	0.27	336.3	346.8
rs141340859		CC/TT	1.65 (0.57-4.80)	0.35	338.3	345.3	1.73 (0.59-5.04)	0.31	335.4	345.8
										
rs5965429		GG/AA	0.75 (0.43-1.30)	0.3	333.9	340.9	0.71 (0.40-1.24)	0.23	331.3	341.8

AIC: Akaike's Information Criterion; BIC: Bayesian Information Criterion; CP/CPPS: chronic prostatitis/chronic pelvic pain syndrome; NA: not applicable; OR: odds ratio; SNPs: single nucleotide polymorphisms. ^a^Adjusted by age.

**Table 4:** Genetic models of SNPs and NIH-CPSI score in patients with CP/CPPS by using logistic regression.

SNP	Model	Genotype	Crude mean difference (95% CI)	*P*	AIC	BIC	Adjusted mean difference (95% CI)^a^	*P*	AIC	BIC
*IFNG*										
rs2069718	Codominant model	GA/AA	1.62 (-1.08-4.31)	0.49	769.2	780.2	1.66 (-1.04-4.36)	0.48	770.3	784.1
		GG/AA	-0.59 (-7.68-6.51)				-0.16 (-7.32-6.99)			
	Log-additive model	-	0.94 (-1.29-3.17)	0.41	768	776.3	1.05 (-1.19-3.29)	0.36	768.9	780
rs1861493	Codominant model	GA/AA	-1.21 (-3.64-1.22)	0.57	769.5	780.6	-1.29 (-3.72-1.15)	0.56	770.6	784.4
		GG/AA	-1.36 (-4.72-2.01)				-1.16 (-4.55-2.23)			
	Log-additive model	-	-0.80 (-2.39-0.80)	0.33	767.7	776	-0.74 (-2.34-0.85)	0.36	768.9	780
*IFNGR1*										
rs10457655	Codominant model	GA/GG	0.98 (-1.71-3.67)	0.1	765.9	777	0.99 (-1.72-3.69)	0.13	767.6	781.5
		AA/GG	9.10 (0.58-17.62)				8.62 (-0.09-17.34)			
	Log-additive model	-	1.95 (-0.41-4.30)	0.11	766	774.3	1.86 (-0.51-4.23)	0.13	767.4	778.4
rs35860242	Codominant model	CT/CC	-0.81 (-4.28-2.65)	0.87	770.4	781.4	-1.05 (-4.54-2.44)	0.77	771.2	785.1
		TT/CC	-1.05 (-8.17-6.06)				-1.67 (-8.88-5.54)			
	Log-additive model	-	-0.67 (-3.22-1.87)	0.6	768.4	776.7	-0.95 (-3.54-1.65)	0.48	769.3	780.3
rs3799488	Codominant model	CT/TT	-0.62 (-2.98-1.73)	0.83	770.3	781.3	-0.48 (-2.86-1.90)	0.88	771.5	785.4
		CC/TT	0.54 (-4.29-5.36)				0.54 (-4.29-5.37)			
	Log-additive model	-	-0.19 (-2.03-1.65)	0.84	768.6	776.9	-0.11 (-1.96-1.73)	0.9	769.8	780.8
rs7749390	Codominant model	GA/GG	0.99 (-1.53-3.51)	0.72	770	781	1.07 (-1.46-3.60)	0.7	771	784.9
		AA/GG	0.21 (-3.02-3.44)				0.36 (-2.89-3.61)			
	Log-additive model	-	0.23 (-1.35-1.81)	0.78	768.6	776.9	0.31 (-1.28-1.90)	0.71	769.6	780.7
rs9376267	Codominant model	CT/CC	-1.21 (-3.74-1.32)	0.56	760.9	771.9	-1.16 (-3.70-1.37)	0.54	762.2	776
		TT/CC	-1.61 (-5.03-1.81)				-1.74 (-5.18-1.69)			
	Log-additive model	-	-0.87 (-2.53-0.79)	0.3	759	767.3	-0.92 (-2.59-0.74)	0.28	760.2	771.3
rs9376268	Codominant model	GA/GG	-1.28 (-3.74-1.18)	0.54	760.8	771.9	-1.27 (-3.73-1.19)	0.52	762.1	775.9
		AA/GG	-1.46 (-4.95-2.02)				-1.66 (-5.19-1.86)			
	Log-additive model	-	-0.85 (-2.51-0.81)	0.32	759	767.3	-0.93 (-2.60-0.74)	0.28	760.2	771.3
*AR*										
rs138464995		TT/CC	1.81 (-3.14-6.76)	0.47	697.9	706	1.65 (-3.35-6.66)	0.52	699.6	710.4
rs142203174		CC/AA	-1.50 (-5.18-2.18)	0.43	768	776.3	-1.26 (-4.99-2.46)	0.51	769.3	780.4
rs192195540		CC/GG	0.97 (-1.87-3.80)	0.51	759.6	767.9	0.92 (-1.92-3.76)	0.53	761	772.1
rs139374285		TT/CC	-1.53 (-7.06-4.00)	0.59	768.3	776.7	-1.56 (-7.09-3.97)	0.58	769.5	780.5
rs72627670		TT/CC	0.02 (-3.21-3.25)	0.99	760.1	768.4	0.07 (-3.17-3.31)	0.97	761.4	772.5
rs144488434		TT/CC	3.69 (-1.80-9.19)	0.19	766.9	775.2	3.55 (-1.96-9.06)	0.21	768.2	779.2
rs143099096		AA/CC	2.57 (-3.57-8.72)	0.41	768	776.3	2.36 (-3.81-8.53)	0.45	769.2	780.3
rs78445514		CC/TT	-0.50 (-3.53-2.53)	0.75	768.5	776.8	-0.74 (-3.80-2.33)	0.64	769.5	780.6
rs141340859		CC/TT	1.98 (-2.17-6.13)	0.35	759.2	767.5	2.02 (-2.14-6.17)	0.34	760.5	771.6
rs5965429		GG/AA	1.51 (-0.98-4.01)	0.24	744.6	752.9	1.51 (-0.99-4.01)	0.24	745.9	756.9

AIC: Akaike's Information Criterion; BIC: Bayesian Information Criterion; CP/CPPS, chronic prostatitis/chronic pelvic pain syndrome; MD: mean difference; NIH-CPSI: National Institutes of Health Chronic Prostatitis Symptom Index; OR: odds ratio; SNPs: single nucleotide polymorphisms. ^a^Adjusted by age.

## Data Availability

The data in the current study are available from the corresponding author on reasonable request.

## Supplementary Materials

Supplementary Materials See Supplemental Figures 1-3 and Supplemental Tables 1-12 in the Supplementary Materials for comprehensive analysis.
