# Supplementary material for: Genetic Polymorphisms of IFNG, IFNGR1, and Androgen Receptor and Chronic Prostatitis/Chronic Pelvic Pain Syndrome in a Chinese Han Population
Source: Dis Markers. 2021 Oct 4;2021:2898336. doi: 10.1155/2021/2898336 (PMC8505099; doi:10.1155/2021/2898336)
Supplement: Supplementary Materials — See Supplemental Figures 1-3 and Supplemental Tables 1-12 in the Supplementary Materials for comprehensive analysis. [file 2898336.f1.docx]

**Genetic Polymorphisms of *IFNG*, *IFNGR1,* and Androgen Receptor and Chronic Prostatitis/Chronic Pelvic Pain Syndrome in a Chinese Han Population**

Lei Chen^1, 2, 3#^, Junyi Chen^1, 2, 3#^, Fan Mo^1, 2, 3^, Zichen Bian^1, 2, 3^, Chen Jin^1, 2, 3^, Xianguo Chen^1, 2, 3^*, Chaozhao Liang^1, 2, 3^*

^1^Department of Urology, The First Affiliated Hospital of Anhui Medical University, Hefei 230022, Anhui, P.R. China

^2^Institute of Urology, Anhui Medical University, Hefei 230022, Anhui, P.R. China

^3^Anhui Province Key Laboratory of Genitourinary Diseases, Anhui Medical University, Hefei 230022, Anhui, P.R. China.

#These authors contributed equally to the work.

**Correspondence to** Xianguo Chen ([cxg7866186@126.com](mailto:cxg7866186@126.com)) & Chaozhao Liang ([liang_chaozhao@ahmu.edu.cn](mailto:liang_chaozhao@ahmu.edu.cn))

**Tel.:** +86 19955195611, and **Fax.:** +86 19955195611

**Address:** Jixi Road 218, Shushan District, Hefei City 230022, Anhui Province, People’s Republic of China.

**Supplemental Figure 1. The association between the 18 genotyped SNPs of *IFNG*, *IFNGR1*, and androgen receptor (*AR*) and NIH-CPSI scores in CP/CPPS patients.** No significant association between the 18 genotyped SNPs and NIH-CPSI scores was found in CP/CPPS patients (**A-R**). The Mann-Whitney U test and Kruskal-Wallis test were applied to analyze the non-normally distributed data between two and three groups, respectively.

**
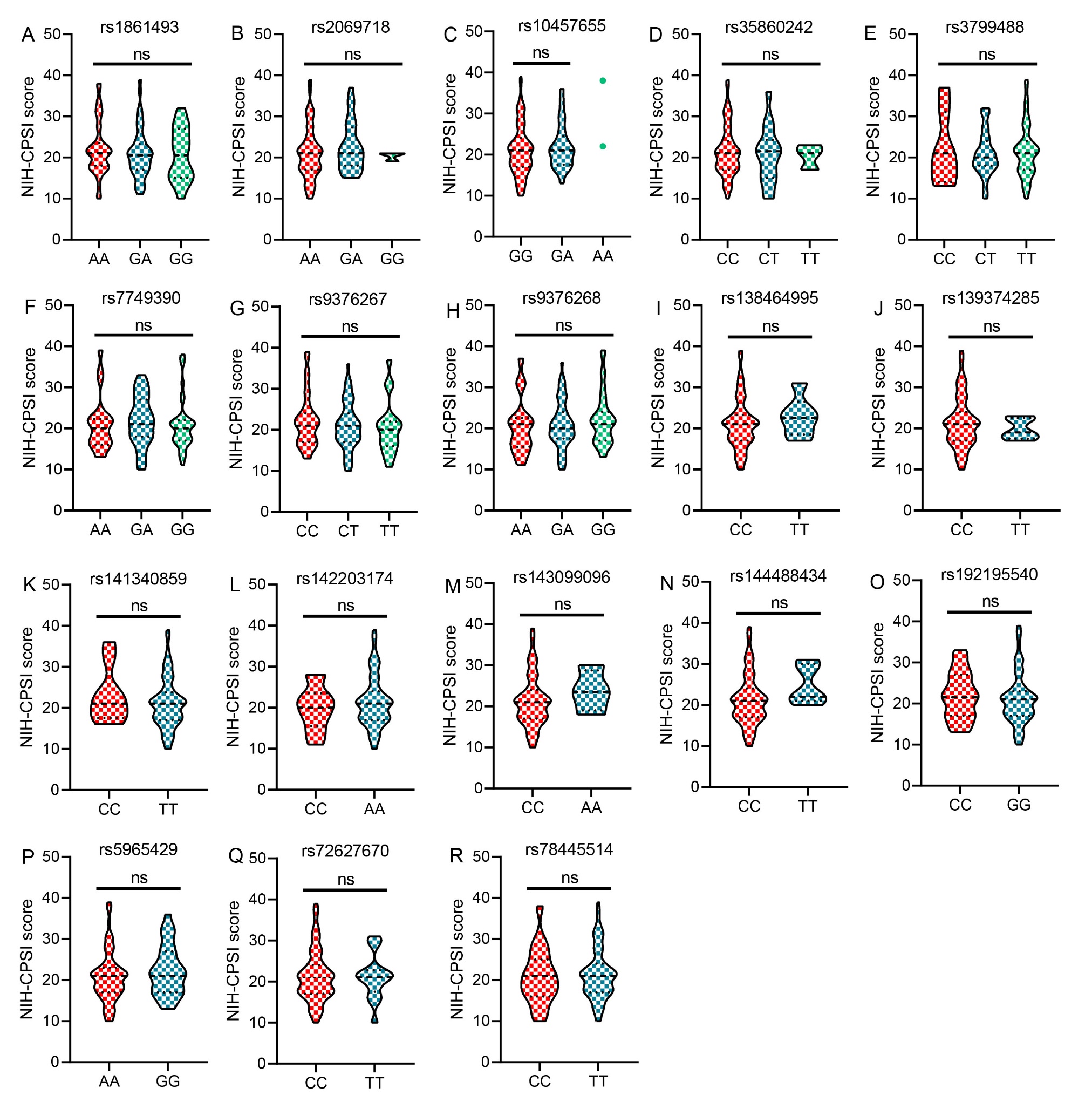
**

CP/CPPS: Chronic prostatitis/chronic pelvic pain syndrome; NIH-CPSI: National Institutes of Health Chronic Prostatitis Symptom Index; SNPs: single nucleotide polymorphisms;

**Supplemental Figure 2. The effects of the SNPs on the expression of their corresponding genes in whole blood.** The results of the GTEx database analysis revealed that rs1861493 (**A**), rs2069718 (**B**), rs7749390 (**D**), rs9376267 (**E**), and rs9376268 (**F**) might affect the expression of their corresponding genes, while rs3799488 (**C**), rs10457655 (**G**), and rs35860242 (**H**) did not affect the expression of the corresponding genes.

**
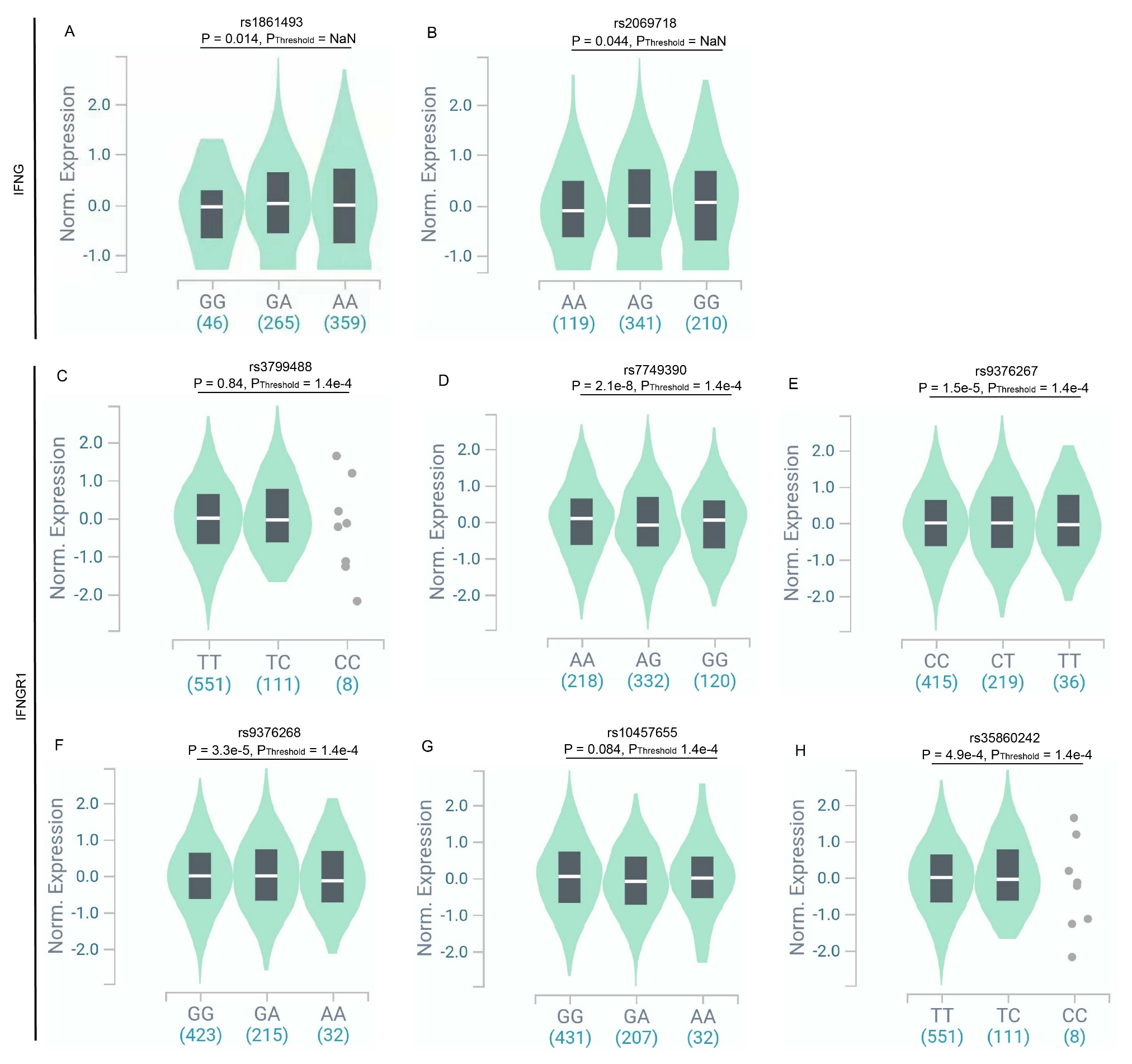
**

SNPs: single nucleotide polymorphisms;

**Supplemental Figure 3. The alterations of serum IFN-γ levels in CP/CPPS patients and healthy controls.** The serum IFN-γ levels were not elevated in CP/CPPS patients compared to healthy controls (**A**), and the genotypes of rs1861493 (**B**) and rs2069718 (**C**) were not associated with the serum IFN-γ levels in CP/CPPS patients. Data were presented as median (Q1, Q3) in the figure, and the Mann-Whitney U test was applied to analyze the non-normally distributed data.

**
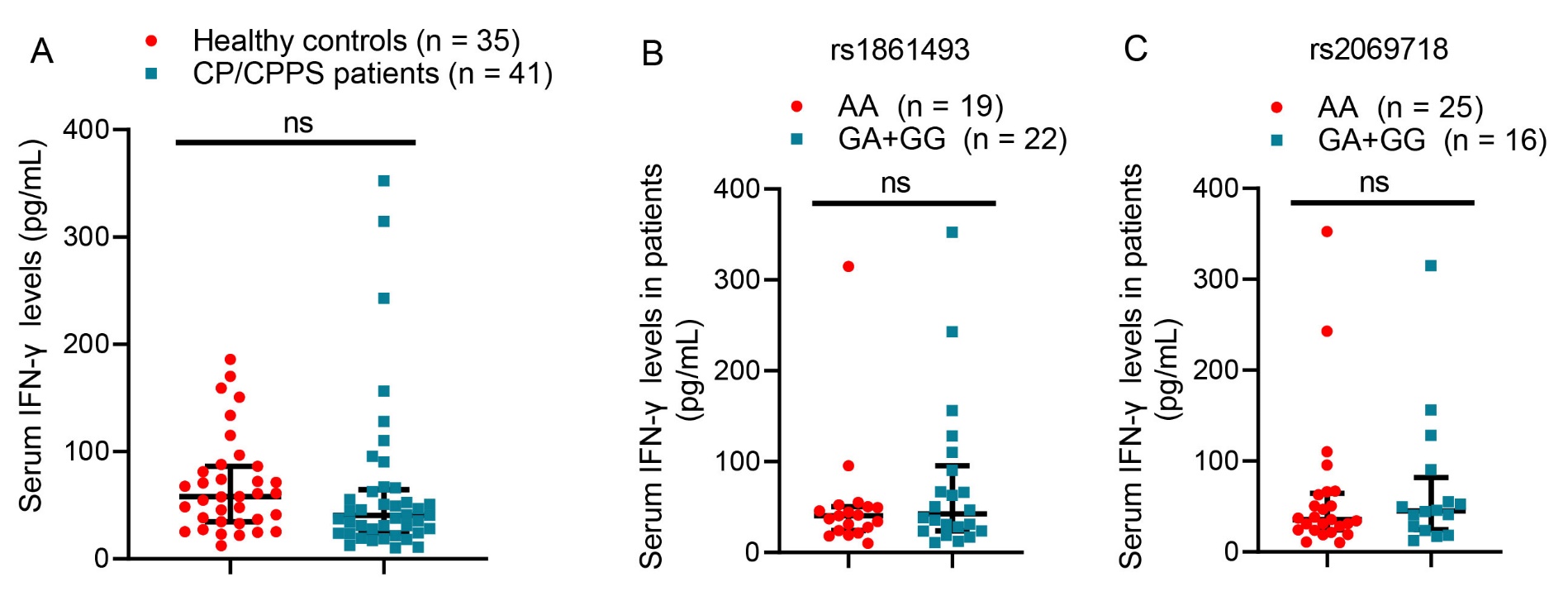
**

**Supplemental Table 1. Basic information of the SNPs in this study.**

| SNP | Chr. | Chr. Position | Gene | Ref mRNA | SNP Property | Length | Alleles | MAF | *P*_HWE_ |
| --- | --- | --- | --- | --- | --- | --- | --- | --- | --- |
| rs1861493 | 12 | 68551196 | *IFNG* | NM_000619 | intron3 | 207 | A/G | 0.383 | 0.5807 |
| rs2069718 | 12 | 68550162 | *IFNG* | NM_000619 | intron3 | 286 | A/G | 0.119 | 0.9063 |
| rs7749390 | 6 | 137540370 | *IFNGR1* | NM_000416 | intron1 | 290 | G/A | 0.432 | 0.8456 |
| rs9376267 | 6 | 137531031 | *IFNGR1* | NM_000416 | intron1 | 103 | C/T | 0.440 | 0.5580 |
| rs10457655 | 6 | 137539810 | *IFNGR1* | NM_000416 | intron1 | 136 | G/A | 0.113 | 0.7647 |
| rs3799488 | 6 | 137519780 | *IFNGR1* | NM_000416 | intron6 | 216 | T/C | 0.253 | 0.3029 |
| rs35860242 | 6 | 137521772 | *IFNGR1* | NM_000416 | intron6 | 221 | C/T | 0.068 | 0.1681 |
| rs9376268 | 6 | 137532751 | *IFNGR1* | NM_000416 | intron1 | 343 | G/A | 0.407 | 0.5106 |
| rs192195540 | X | 66791836 | *AR* | NM_000044 | intron1 | 308 | G/C | 0.211 | NA |
| rs139374285 | X | 66914636 | *AR* | NM_000044 | intron3 | 400 | C/T | 0.045 | NA |
| rs72627670 | X | 66892219 | *AR* | NM_000044 | intron2 | 213 | C/T | 0.165 | NA |
| rs144488434 | X | 66891664 | *AR* | NM_000044 | intron2 | 290 | C/T | 0.070 | NA |
| rs138464995 | X | 66799594 | *AR* | NM_000044 | intron1 | 406 | C/T | 0.052 | NA |
| rs143099096 | X | 66875577 | *AR* | NM_000044 | intron2 | 352 | C/A | 0.037 | NA |
| rs78445514 | X | 66841609 | *AR* | NM_000044 | intron1 | 160 | T/C | 0.136 | NA |
| rs141340859 | X | 66898794 | *AR* | NM_000044 | intron2 | 106 | T/C | 0.062 | NA |
| rs142203174 | X | 66855153 | *AR* | NM_000044 | intron1 | 247 | A/C | 0.091 | NA |
| rs5965429 | X | 66828653 | *AR* | NM_000044 | intron1 | 303 | A/G | 0.301 | NA |

Chr: chromosome; HWE: Hardy-Weinberg equilibrium; MAF, minor allele frequency; NA, not applicable.

**Supplemental Table 2. The primers used in this study.**

| Gene | SNP |  | Primer sequence (5'‑3') |
| --- | --- | --- | --- |
| *IFNG* | rs2069718 | Forward | ACAACTTTTCCAGTACCCTGCCTTC |
|  |  | Reverse | GAAATGTGGTGAGTAGCCATAGTGTTC |
|  | rs1861493 | Forward | AGAGACTTGCAGTGGGGTGTCC |
|  |  | Reverse | CTCAGCCACCAAGGAACTGTGA |
| *IFNGR1* | rs10457655 | Forward | AGCCACGAACTGAGACCACGAT |
|  |  | Reverse | GTCTGGGTTTCTACTGCCCGAAG |
|  | rs35860242 | Forward | ACAAAGTCCTCATGGGCTCAGGT |
|  |  | Reverse | CCTGCCATCAATCAGTCAGTCTACC |
|  | rs3799488 | Forward | GACAACGGCTCTTCACAGACCA |
|  |  | Reverse | CCATTTGGTGGTCCATTACTTCAGA |
|  | rs7749390 | Forward | AAAGGCAACCGACGAGTTCAAA |
|  |  | Reverse | AGCAGCATGGCTCTCCTCTTTC |
|  | rs9376267 | Forward | GATTGAACAATGGAGCCACACATTC |
|  |  | Reverse | GTGAAGGCAAGAAGAAATGTTGGGTAT |
|  | rs9376268 | Forward | TCATGGCTCCCCTCAAACAAAT |
|  |  | Reverse | GAATGGCCATGGGAACTCTACAA |
| *AR* | rs138464995 | Forward | TTCCCTGTCCTCCTAGGCACAT |
|  |  | Reverse | TGGCTGAGGAAAGGCCAAAATA |
|  | rs142203174 | Forward | TTAAGGCAACCAAGACTTACAATGTGC |
|  |  | Reverse | CAAGAGGAATGGCAACCCAACA |
|  | rs192195540 | Forward | ACATTTGTATGTGGGCTCTACATTGC |
|  |  | Reverse | CCCCAGAAAAGGCAGAATCAGA |
|  | rs139374285 | Forward | GCCCACCTCCCCAACTTTACAT |
|  |  | Reverse | TCTACAGCCCGTCCTCACAACA |
|  | rs72627670 | Forward | CTCCAGCTCCATCCAGGTGTCT |
|  |  | Reverse | CACCACCAGAGAAAACCACTTTCA |
|  | rs144488434 | Forward | TTGTATAACGTGTAAGGAAGAGGTCCAC |
|  |  | Reverse | GAGATATAGACACGTGGAACAGAGCAG |
|  | rs143099096 | Forward | TGGAGGTATAGAGTGATTTCCCACCTAC |
|  |  | Reverse | GTGCTGTAATTTGATGAGGGACTGATT |
|  | rs78445514 | Forward | TGCTCACTCCAGTTCAATTAAATCAG |
|  |  | Reverse | TCAAAGTTGGATGGCCCTTATCA |
|  | rs141340859 | Forward | GGAGGCAGTTGACTGAGCTAGGAA |
|  |  | Reverse | TGGTCACCATGCTGTGCAATAG |
|  | rs5965429 | Forward | TGAAATTCAGACCTGCAATGAAGTG |
|  |  | Reverse | AATATTGGAAGGGTATGCCAAGAAGC |

**Supplemental Table 3. Predicted function of these 18 SNPs by RegulomeDB and HaploReg.**

| SNP | RegulomeDB |  | HaploReg | | | | |
| --- | --- | --- | --- | --- | --- | --- | --- |
|  | RegulomeDB score |  | Histone marker | DNase | Protein bound | Motifs changed | eQTL hits |
| rs1861493 | 5 |  | + | *+* | - | - | *+* |
| rs2069718 | 7 |  | + | *+* | - | - | *+* |
| rs7749390 | 4 |  | + | *+* | *+* | *+* | *+* |
| rs9376267 | 4 |  | + | *-* | - | *+* | *+* |
| rs10457655 | 2b |  | - | *+* | *+* | *+* | - |
| rs3799488 | 3a |  | - | *-* | - | *+* | - |
| rs35860242 | 5 |  | + | *+* | - | *+* | - |
| rs9376268 | 5 |  | + | *+* | - | *+* | *+* |
| rs192195540 | 6 |  | - | *-* | - | *+* | - |
| rs139374285 | 4 |  | + | *+* | - | *+* | - |
| rs72627670 | 7 |  | - | *-* | - | *+* | - |
| rs144488434 | 7 |  | - | *-* | - | *+* | - |
| rs138464995 | 7 |  | + | *-* | - | *+* | - |
| rs143099096 | 5 |  | + | *-* | - | *+* | - |
| rs78445514 | 7 |  | - | *-* | - | *+* | - |
| rs141340859 | 7 |  | - | *-* | - | *+* | - |
| rs142203174 | 5 |  | + | *-* | - | *+* | - |
| rs5965429 | 6 |  | + | *-* | - | - | - |

eQTL: expression quantitative trait loci.

‘

**Supplemental Table 4. Genetic models of SNPs and CP/CPPS in patients < 35 years by using logistic regression.**

| Gene / SNP | Model | Genotype | Crude OR (95%CI) | *P* | AIC | BIC | Adjusted OR (95%CI)^a^ | *P* | AIC | BIC |
| --- | --- | --- | --- | --- | --- | --- | --- | --- | --- | --- |
| *IFNG* |  |  |  |  |  |  |  |  |  |  |
| rs2069718 | Codominant model | GA/AA | 0.65 (0.27, 1.60) | 0.35 | 172.1 | 177.7 | 0.68 (0.28, 1.68) | 0.40 | 170.8 | 179.2 |
| rs1861493 | Codominant model | GA/AA | 1.08 (0.49, 2.35) | 0.90 | 174.8 | 183.2 | 1.12 (0.51, 2.46) | 0.77 | 173 | 184.2 |
|  |  | GG/AA | 1.29 (0.42, 3.99) |  |  |  | 1.54 (0.48, 4.92) |  |  |  |
|  | Log-additive model | - | 1.12 (0.66, 1.91) | 0.67 | 172.8 | 178.4 | 1.21 (0.70, 2.09) | 0.50 | 171 | 179.4 |
| *IFNGR1* |  |  |  |  |  |  |  |  |  |  |
| rs10457655 | Codominant model | GA/GG | 0.92 (0.37, 2.26) | 0.23 | 172 | 180.4 | 0.87 (0.35, 2.18) | 0.36 | 171.4 | 182.7 |
|  | Log-additive model | - | 1.26 (0.58, 2.74) | 0.56 | 172.7 | 178.3 | 1.12 (0.50, 2.52) | 0.78 | 171.4 | 179.8 |
| rs35860242 | Codominant model | CT/CC | 1.58 (0.51, 4.88) | 0.079 | 169.9 | 178.3 | 1.66 (0.53, 5.18) | 0.09 | 168.7 | 179.9 |
|  | Log-additive model | - | 2.28 (0.90, 5.77) | 0.064 | 169.6 | 175.2 | 2.31 (0.89, 5.96) | 0.066 | 168.1 | 176.5 |
| rs3799488 | Codominant model | CT/TT | 1.08 (0.49, 2.39) | 0.95 | 174.9 | 183.3 | 1.33 (0.58, 3.05) | 0.73 | 172.9 | 184.1 |
|  |  | CC/TT | 0.86 (0.22, 3.47) |  |  |  | 0.82 (0.20, 3.40) |  |  |  |
|  | Log-additive model | - | 0.99 (0.56, 1.74) | 0.97 | 173 | 178.6 | 1.06 (0.59, 1.89) | 0.84 | 171.4 | 179.9 |
| rs7749390 | Codominant model | GA/GG | 0.87 (0.38, 1.97) | 0.61 | 174 | 182.4 | 0.96 (0.42, 2.21) | 0.60 | 172.5 | 183.7 |
|  |  | AA/GG | 0.60 (0.22, 1.67) |  |  |  | 0.62 (0.22, 1.74) |  |  |  |
|  | Log-additive model | - | 0.78 (0.47, 1.30) | 0.34 | 172.1 | 177.7 | 0.80 (0.48, 1.34) | 0.39 | 170.8 | 179.2 |
| rs9376267 | Codominant model | CT/CC | 1.14 (0.51, 2.55) | 0.86 | 173.2 | 181.6 | 1.27 (0.56, 2.90) | 0.81 | 171.8 | 182.9 |
|  |  | TT/CC | 1.34 (0.47, 3.88) |  |  |  | 1.35 (0.46, 3.95) |  |  |  |
|  | Log-additive model | - | 1.16 (0.69, 1.94) | 0.58 | 171.2 | 176.8 | 1.18 (0.70, 1.99) | 0.54 | 169.8 | 178.2 |
| rs9376268 | Codominant model | GA/GG | 0.98 (0.44, 2.17) | 0.64 | 172.6 | 181 | 1.09 (0.49, 2.47) | 0.71 | 171.5 | 182.7 |
|  |  | AA/GG | 1.59 (0.53, 4.77) |  |  |  | 1.59 (0.52, 4.84) |  |  |  |
|  | Log-additive model | - | 1.20 (0.71, 2.03) | 0.50 | 171.1 | 176.7 | 1.23 (0.72, 2.10) | 0.45 | 169.6 | 178 |
| *AR* |  |  |  |  |  |  |  |  |  |  |
| rs138464995 |  | TT/CC | 1.57 (0.34, 7.34) | 0.56 | 166.7 | 172.3 | 1.54 (0.33, 7.26) | 0.58 | 165.2 | 173.5 |
| rs142203174 |  | CC/AA | 1.08 (0.33, 3.54) | 0.90 | 173 | 178.6 | 1.11 (0.33, 3.72) | 0.86 | 171.4 | 179.9 |
| rs192195540 |  | CC/GG | 0.62 (0.28, 1.40) | 0.25 | 170.2 | 175.8 | 0.65 (0.29, 1.49) | 0.31 | 169.2 | 177.5 |
| rs139374285 |  | TT/CC | 1.63 (0.26, 10.14) | 0.59 | 172.7 | 178.3 | 1.45 (0.23, 9.18) | 0.69 | 171.3 | 179.7 |
| rs72627670 |  | TT/CC | 0.79 (0.26, 2.44) | 0.69 | 171.4 | 177 | 0.72 (0.22, 2.29) | 0.57 | 169.9 | 178.3 |
| rs144488434 |  | TT/CC | 0.33 (0.06, 1.72) | 0.16 | 171 | 176.6 | 0.23 (0.04, 1.40) | 0.084 | 168.5 | 176.9 |
| rs143099096 |  | AA/CC | 0.84 (0.22, 3.31) | 0.81 | 172.9 | 178.5 | 0.95 (0.24, 3.84) | 0.95 | 171.5 | 179.9 |
| rs78445514 |  | CC/TT | 2.96 (0.97, 9.00) | 0.056 | 169 | 174.6 | 2.94 (0.95, 9.06) | 0.06 | 167.6 | 176.1 |
| rs141340859 |  | CC/TT | 2.26 (0.40, 12.83) | 0.34 | 170.6 | 176.2 | 2.68 (0.46-15.65) | 0.26 | 168.9 | 177.3 |
| rs5965429 |  | GG/AA | 0.59 (0.28-1.28) | 0.18 | 168.2 | 173.8 | 0.64 (0.29-1.39) | 0.26 | 167.2 | 175.6 |

AIC, Akaike’s Information Criterion; BIC, Bayesian Information Criterion; CP/CPPS, chronic prostatitis/chronic pelvic pain syndrome; OR, odds ratio; SNPs, single nucleotide polymorphisms.

^a^Adjusted by age.

**Supplemental Table 5. Genetic models of SNPs and CP/CPPS in patients ≥ 35 years by using logistic regression.**

| Gene / SNP | Model | Genotype | Crude OR (95%CI) | *P* | AIC | BIC | Adjusted OR (95%CI)^a^ | *P* | AIC | BIC |
| --- | --- | --- | --- | --- | --- | --- | --- | --- | --- | --- |
| *IFNG* |  |  |  |  |  |  |  |  |  |  |
| rs2069718 | Codominant model | GA/AA | 2.31 (0.93, 5.77) | 0.10 | 169.1 | 177.5 | 1.64 (0.62, 4.32) | 0.32 | 159.1 | 170.3 |
|  |  | GG/AA | 3.90 (0.39, 38.92) |  |  |  | 3.88 (0.36, 41.60) |  |  |  |
|  | Log-additive model | - | **2.18 (1.03**, **4.64)** | **0.034** | 167.2 | 172.8 | 1.77 (0.82, 3.81) | 0.14 | 157.2 | 165.6 |
| rs1861493 | Codominant model | GA/AA | 1.00 (0.46, 2.19) | 0.84 | 173.3 | 181.7 | 1.08 (0.47, 2.49) | 0.97 | 161.3 | 172.5 |
|  |  | GG/AA | 0.75 (0.27, 2.08) |  |  |  | 0.96 (0.33, 2.83) |  |  |  |
|  | Log-additive model | - | 0.89 (0.55, 1.45) | 0.64 | 171.4 | 177 | 1.00 (0.59, 1.68) | 0.99 | 159.4 | 167.8 |
| *IFNGR1* |  |  |  |  |  |  |  |  |  |  |
| rs10457655 | Codominant model | GA/GG | 1.17 (0.50, 2.76) | 0.72 | 171.5 | 177.1 | 1.04 (0.42, 2.58) | 0.93 | 159.4 | 167.8 |
| rs35860242 | Codominant model | CT/CC | 0.89 (0.28, 2.82) | 0.84 | 171.6 | 177.2 | 0.59 (0.17, 2.00) | 0.40 | 158.7 | 167 |
| rs3799488 | Codominant model | CT/TT | 1.21 (0.57, 2.55) | 0.40 | 171.8 | 180.2 | 1.13 (0.51, 2.51) | 0.48 | 159.9 | 171.1 |
|  |  | CC/TT | 0.46 (0.11, 1.93) |  |  |  | 0.46 (0.10, 2.03) |  |  |  |
|  | Log-additive model | - | 0.88 (0.50, 1.53) | 0.64 | 171.4 | 177 | 0.84 (0.47, 1.52) | 0.57 | 159.1 | 167.4 |
| rs7749390 | Codominant model | GA/GG | 0.84 (0.38, 1.88) | 0.36 | 171.6 | 180 | 0.95 (0.40, 2.21) | 0.23 | 158.4 | 169.6 |
|  |  | AA/GG | 1.80 (0.59, 5.55) |  |  |  | 2.45 (0.74, 8.19) |  |  |  |
|  | Log-additive model | - | 1.22 (0.72, 2.08) | 0.46 | 171.1 | 176.7 | 1.41 (0.80, 2.49) | 0.23 | 157.9 | 166.3 |
| rs9376267 | Codominant model | CT/CC | 1.03 (0.45, 2.39) | 0.32 | 171.4 | 179.8 | 0.93 (0.38, 2.28) | 0.18 | 158 | 169.2 |
|  |  | TT/CC | 0.50 (0.17, 1.50) |  |  |  | 0.38 (0.12, 1.23) |  |  |  |
|  | Log-additive model | - | 0.74 (0.43, 1.26) | 0.27 | 170.4 | 176 | 0.64 (0.36, 1.14) | 0.13 | 157.1 | 165.4 |
| rs9376268 | Codominant model | GA/GG | 1.19 (0.54, 2.63) | 0.33 | 171.4 | 179.8 | 0.99 (0.42, 2.31) | 0.26 | 158.7 | 169.8 |
|  |  | AA/GG | 0.53 (0.17, 1.67) |  |  |  | 0.40 (0.12, 1.36) |  |  |  |
|  | Log-additive model | - | 0.81 (0.48, 1.39) | 0.45 | 171.1 | 176.7 | 0.70 (0.39, 1.24) | 0.21 | 157.8 | 166.2 |
| *AR* |  |  |  |  |  |  |  |  |  |  |
| rs138464995 |  | TT/CC | 0.77 (0.12, 4.80) | 0.78 | 162.6 | 168.1 | 0.49 (0.07, 3.43) | 0.47 | 151.4 | 159.7 |
| rs142203174 |  | CC/AA | 1.64 (0.44, 6.14) | 0.46 | 171.1 | 176.7 | 3.28 (0.75, 14.28) | 0.10 | 156.7 | 165.1 |
| rs192195540 |  | CC/GG | 1.06 (0.39, 2.89) | 0.91 | 171.7 | 177.2 | 0.92 (0.32, 2.62) | 0.88 | 159.4 | 167.7 |
| rs139374285 |  | TT/CC | 0.51 (0.09, 2.89) | 0.43 | 171.1 | 176.6 | 0.57 (0.09, 3.56) | 0.54 | 159 | 167.4 |
| rs72627670 |  | TT/CC | 0.59 (0.24, 1.42) | 0.23 | 170.2 | 175.8 | 0.49 (0.19, 1.29) | 0.14 | 157.2 | 165.6 |
| rs144488434 |  | TT/CC | 0.50 (0.12, 2.10) | 0.33 | 170.7 | 176.3 | 0.33 (0.07, 1.56) | 0.15 | 157.3 | 165.7 |
| rs78445514 |  | CC/TT | 0.79 (0.27, 2.29) | 0.67 | 171.5 | 177.1 | 0.81 (0.27, 2.46) | 0.71 | 159.2 | 167.6 |
| rs141340859 |  | CC/TT | 1.34 (0.34, 5.26) | 0.67 | 171.5 | 177.1 | 1.11 (0.27, 4.48) | 0.89 | 159.4 | 167.7 |
| rs5965429 |  | GG/AA | 0.98 (0.43, 2.23) | 0.96 | 168.9 | 174.4 | 0.84 (0.36, 1.98) | 0.69 | 158 | 166.4 |

AIC, Akaike’s Information Criterion; BIC, Bayesian Information Criterion; CP/CPPS, chronic prostatitis/chronic pelvic pain syndrome; OR, odds ratio; SNPs, single nucleotide polymorphisms.

^a^Adjusted by age

**Supplemental Table 6. *IFNGR1* and *AR* haplotype frequencies and CP/CPPS.**

| Gene | Combinations of SNPs | Haplotype | Case, n (%) | Control, n(%) | OR (95% CI) | χ^2^ | *P* |
| --- | --- | --- | --- | --- | --- | --- | --- |
| *IFNGR1* | rs9376267-rs9376268 | CG | 133 (56.8) | 138 (55.2) | 1.07 (0.75, 1.53) | 0.132 | 0.717 |
|  |  | TA | 95 (40.6) | 102 (40.8) | 0.99 (0.69, 1.43) | 0.002 | 0.964 |
|  |  | TG | 6 (2.60) | 10 (4.00) | 0.63 (0.23, 1.77) | 0.780 | 0.377 |
| *AR* | rs5965429-rs192195540 | AG | 168 (73.0) | 166 (66.9) | 1.34 (0.90, 1.98) | 2.115 | 0.146 |
|  |  | GC | 44 (19.1) | 58 (23.4) | 0.78 (0.50, 1.20) | 1.288 | 0.256 |
|  |  | GG | 18 (7.8) | 24 (9.7) | 0.79 (0.42, 1.50) | 0.510 | 0.475 |

CP/CPPS, chronic prostatitis/chronic pelvic pain syndrome; OR, odds ratio; SNPs, single nucleotide polymorphisms.

| **Supplemental Table 7. The rs1861493 associated and interacted single nucleotide polymorphisms (SNPs) through three-dimensional (3D) chromatin looping (LD r^2^ > 0.8).** | | | | | | | | |
| --- | --- | --- | --- | --- | --- | --- | --- | --- |
| **SNP ID** | **Population** | **LD(r2)** | **Loop type** | **Loop start** | **Loop end** | **Distance** | **Cell type** | **Tissue** |
| rs10083034 | AMR | 0.98633 | Within loop | chr12:68400000-68410000 | chr12:68580000-68590000 | 180000 | H1-hESC | ESC |
| rs10083034 | EAS | 0.97205 | Within loop | chr12:68400000-68410000 | chr12:68580000-68590000 | 180000 | H1-hESC | ESC |
| rs10083034 | EUR | 0.98494 | Within loop | chr12:68400000-68410000 | chr12:68580000-68590000 | 180000 | H1-hESC | ESC |
| rs10083034 | SAS | 0.97633 | Within loop | chr12:68400000-68410000 | chr12:68580000-68590000 | 180000 | H1-hESC | ESC |
| rs10083165 | AMR | 0.98633 | Within loop | chr12:68400000-68410000 | chr12:68580000-68590000 | 180000 | H1-hESC | ESC |
| rs10083165 | EAS | 0.97205 | Within loop | chr12:68400000-68410000 | chr12:68580000-68590000 | 180000 | H1-hESC | ESC |
| rs10083165 | EUR | 0.98494 | Within loop | chr12:68400000-68410000 | chr12:68580000-68590000 | 180000 | H1-hESC | ESC |
| rs10083165 | SAS | 0.97633 | Within loop | chr12:68400000-68410000 | chr12:68580000-68590000 | 180000 | H1-hESC | ESC |
| rs10161291 | AMR | 0.98633 | Within loop | chr12:68400000-68410000 | chr12:68580000-68590000 | 180000 | H1-hESC | ESC |
| rs10161291 | EAS | 0.97205 | Within loop | chr12:68400000-68410000 | chr12:68580000-68590000 | 180000 | H1-hESC | ESC |
| rs10161291 | EUR | 0.98494 | Within loop | chr12:68400000-68410000 | chr12:68580000-68590000 | 180000 | H1-hESC | ESC |
| rs10161291 | SAS | 0.97633 | Within loop | chr12:68400000-68410000 | chr12:68580000-68590000 | 180000 | H1-hESC | ESC |
| rs10748098 | AMR | 0.94684 | Within loop | chr12:68400000-68410000 | chr12:68580000-68590000 | 180000 | H1-hESC | ESC |
| rs10748098 | EUR | 0.9413 | Within loop | chr12:68400000-68410000 | chr12:68580000-68590000 | 180000 | H1-hESC | ESC |
| rs10748098 | SAS | 0.922 | Within loop | chr12:68400000-68410000 | chr12:68580000-68590000 | 180000 | H1-hESC | ESC |
| rs10748099 | EUR | 0.9606 | Within loop | chr12:68400000-68410000 | chr12:68580000-68590000 | 180000 | H1-hESC | ESC |
| rs10748099 | SAS | 0.83731 | Within loop | chr12:68400000-68410000 | chr12:68580000-68590000 | 180000 | H1-hESC | ESC |
| rs10784679 | AMR | 0.98633 | Within loop | chr12:68400000-68410000 | chr12:68580000-68590000 | 180000 | H1-hESC | ESC |
| rs10784679 | EAS | 0.97205 | Within loop | chr12:68400000-68410000 | chr12:68580000-68590000 | 180000 | H1-hESC | ESC |
| rs10784679 | EUR | 0.98494 | Within loop | chr12:68400000-68410000 | chr12:68580000-68590000 | 180000 | H1-hESC | ESC |
| rs10784679 | SAS | 0.97633 | Within loop | chr12:68400000-68410000 | chr12:68580000-68590000 | 180000 | H1-hESC | ESC |
| rs10784683 | AMR | 0.94047 | Within loop | chr12:68400000-68410000 | chr12:68580000-68590000 | 180000 | H1-hESC | ESC |
| rs10784683 | EUR | 0.98003 | Within loop | chr12:68400000-68410000 | chr12:68580000-68590000 | 180000 | H1-hESC | ESC |
| rs10784683 | SAS | 0.94488 | Within loop | chr12:68400000-68410000 | chr12:68580000-68590000 | 180000 | H1-hESC | ESC |
| rs10784684 | AMR | 0.94684 | Within loop | chr12:68400000-68410000 | chr12:68580000-68590000 | 180000 | H1-hESC | ESC |
| rs10784684 | EUR | 0.98003 | Within loop | chr12:68400000-68410000 | chr12:68580000-68590000 | 180000 | H1-hESC | ESC |
| rs10784684 | SAS | 0.96056 | Within loop | chr12:68400000-68410000 | chr12:68580000-68590000 | 180000 | H1-hESC | ESC |
| rs10784688 | AMR | 0.93416 | Within loop | chr12:68400000-68410000 | chr12:68580000-68590000 | 180000 | H1-hESC | ESC |
| rs10784688 | EUR | 0.98003 | Within loop | chr12:68400000-68410000 | chr12:68580000-68590000 | 180000 | H1-hESC | ESC |
| rs10784688 | SAS | 0.87482 | Within loop | chr12:68400000-68410000 | chr12:68580000-68590000 | 180000 | H1-hESC | ESC |
| rs10878763 | AMR | 0.98633 | Within loop | chr12:68400000-68410000 | chr12:68580000-68590000 | 180000 | H1-hESC | ESC |
| rs10878763 | EAS | 1 | Within loop | chr12:68400000-68410000 | chr12:68580000-68590000 | 180000 | H1-hESC | ESC |
| rs10878763 | EUR | 1 | Within loop | chr12:68400000-68410000 | chr12:68580000-68590000 | 180000 | H1-hESC | ESC |
| rs10878763 | SAS | 0.9842 | Within loop | chr12:68400000-68410000 | chr12:68580000-68590000 | 180000 | H1-hESC | ESC |
| rs10878779 | AMR | 0.9095 | Within loop | chr12:68400000-68410000 | chr12:68580000-68590000 | 180000 | H1-hESC | ESC |
| rs10878779 | EUR | 0.97512 | Within loop | chr12:68400000-68410000 | chr12:68580000-68590000 | 180000 | H1-hESC | ESC |
| rs10878779 | SAS | 0.87482 | Within loop | chr12:68400000-68410000 | chr12:68580000-68590000 | 180000 | H1-hESC | ESC |
| rs1115577 | AMR | 0.95326 | Within loop | chr12:68400000-68410000 | chr12:68580000-68590000 | 180000 | H1-hESC | ESC |
| rs1115577 | EAS | 1 | Within loop | chr12:68400000-68410000 | chr12:68580000-68590000 | 180000 | H1-hESC | ESC |
| rs1115577 | EUR | 0.99499 | Within loop | chr12:68400000-68410000 | chr12:68580000-68590000 | 180000 | H1-hESC | ESC |
| rs1115577 | SAS | 0.97633 | Within loop | chr12:68400000-68410000 | chr12:68580000-68590000 | 180000 | H1-hESC | ESC |
| rs1118865 | AMR | 0.98633 | Within loop | chr12:68400000-68410000 | chr12:68580000-68590000 | 180000 | H1-hESC | ESC |
| rs1118865 | EAS | 0.98603 | Within loop | chr12:68400000-68410000 | chr12:68580000-68590000 | 180000 | H1-hESC | ESC |
| rs1118865 | EUR | 0.98494 | Within loop | chr12:68400000-68410000 | chr12:68580000-68590000 | 180000 | H1-hESC | ESC |
| rs1118865 | SAS | 0.97633 | Within loop | chr12:68400000-68410000 | chr12:68580000-68590000 | 180000 | H1-hESC | ESC |
| rs1118866 | AMR | 0.8975 | Within loop | chr12:68400000-68410000 | chr12:68580000-68590000 | 180000 | H1-hESC | ESC |
| rs1118866 | EAS | 0.98603 | Within loop | chr12:68400000-68410000 | chr12:68580000-68590000 | 180000 | H1-hESC | ESC |
| rs1118866 | EUR | 0.97995 | Within loop | chr12:68400000-68410000 | chr12:68580000-68590000 | 180000 | H1-hESC | ESC |
| rs1118866 | SAS | 0.96842 | Within loop | chr12:68400000-68410000 | chr12:68580000-68590000 | 180000 | H1-hESC | ESC |
| rs112212239 | EUR | 0.97025 | Within loop | chr12:68400000-68410000 | chr12:68580000-68590000 | 180000 | H1-hESC | ESC |
| rs112212239 | SAS | 0.83686 | Within loop | chr12:68400000-68410000 | chr12:68580000-68590000 | 180000 | H1-hESC | ESC |
| rs11390256 | AMR | 0.93416 | Within loop | chr12:68400000-68410000 | chr12:68580000-68590000 | 180000 | H1-hESC | ESC |
| rs11390256 | EUR | 0.98003 | Within loop | chr12:68400000-68410000 | chr12:68580000-68590000 | 180000 | H1-hESC | ESC |
| rs11390256 | SAS | 0.87482 | Within loop | chr12:68400000-68410000 | chr12:68580000-68590000 | 180000 | H1-hESC | ESC |
| rs11458654 | AMR | 1 | Within loop | chr12:68400000-68410000 | chr12:68580000-68590000 | 180000 | H1-hESC | ESC |
| rs11458654 | EUR | 0.98498 | Within loop | chr12:68400000-68410000 | chr12:68580000-68590000 | 180000 | H1-hESC | ESC |
| rs11458654 | SAS | 0.96842 | Within loop | chr12:68400000-68410000 | chr12:68580000-68590000 | 180000 | H1-hESC | ESC |
| rs1861494 | AMR | 0.93416 | Within loop | chr12:68400000-68410000 | chr12:68580000-68590000 | 180000 | H1-hESC | ESC |
| rs1861494 | EAS | 0.98141 | Within loop | chr12:68400000-68410000 | chr12:68580000-68590000 | 180000 | H1-hESC | ESC |
| rs1861494 | EUR | 0.99499 | Within loop | chr12:68400000-68410000 | chr12:68580000-68590000 | 180000 | H1-hESC | ESC |
| rs1861494 | SAS | 0.99214 | Within loop | chr12:68400000-68410000 | chr12:68580000-68590000 | 180000 | H1-hESC | ESC |
| rs2041863 | AMR | 0.98633 | Within loop | chr12:68400000-68410000 | chr12:68580000-68590000 | 180000 | H1-hESC | ESC |
| rs2041863 | EAS | 0.97209 | Within loop | chr12:68400000-68410000 | chr12:68580000-68590000 | 180000 | H1-hESC | ESC |
| rs2041863 | EUR | 0.98997 | Within loop | chr12:68400000-68410000 | chr12:68580000-68590000 | 180000 | H1-hESC | ESC |
| rs2041863 | SAS | 0.9842 | Within loop | chr12:68400000-68410000 | chr12:68580000-68590000 | 180000 | H1-hESC | ESC |
| rs2098394 | AMR | 0.94684 | Within loop | chr12:68400000-68410000 | chr12:68580000-68590000 | 180000 | H1-hESC | ESC |
| rs2098394 | EUR | 0.98003 | Within loop | chr12:68400000-68410000 | chr12:68580000-68590000 | 180000 | H1-hESC | ESC |
| rs2098394 | SAS | 0.96056 | Within loop | chr12:68400000-68410000 | chr12:68580000-68590000 | 180000 | H1-hESC | ESC |
| rs2111059 | AMR | 0.98633 | Within loop | chr12:68400000-68410000 | chr12:68580000-68590000 | 180000 | H1-hESC | ESC |
| rs2111059 | EAS | 0.99533 | Within loop | chr12:68400000-68410000 | chr12:68580000-68590000 | 180000 | H1-hESC | ESC |
| rs2111059 | EUR | 1 | Within loop | chr12:68400000-68410000 | chr12:68580000-68590000 | 180000 | H1-hESC | ESC |
| rs2111059 | SAS | 0.97633 | Within loop | chr12:68400000-68410000 | chr12:68580000-68590000 | 180000 | H1-hESC | ESC |
| rs2193045 | AMR | 0.92172 | Within loop | chr12:68400000-68410000 | chr12:68580000-68590000 | 180000 | H1-hESC | ESC |
| rs2193045 | EAS | 0.97205 | Within loop | chr12:68400000-68410000 | chr12:68580000-68590000 | 180000 | H1-hESC | ESC |
| rs2193045 | EUR | 0.98495 | Within loop | chr12:68400000-68410000 | chr12:68580000-68590000 | 180000 | H1-hESC | ESC |
| rs2193045 | SAS | 0.9842 | Within loop | chr12:68400000-68410000 | chr12:68580000-68590000 | 180000 | H1-hESC | ESC |
| rs2193048 | AMR | 0.95326 | Within loop | chr12:68400000-68410000 | chr12:68580000-68590000 | 180000 | H1-hESC | ESC |
| rs2193048 | EAS | 0.97205 | Within loop | chr12:68400000-68410000 | chr12:68580000-68590000 | 180000 | H1-hESC | ESC |
| rs2193048 | EUR | 0.98495 | Within loop | chr12:68400000-68410000 | chr12:68580000-68590000 | 180000 | H1-hESC | ESC |
| rs2193048 | SAS | 0.9842 | Within loop | chr12:68400000-68410000 | chr12:68580000-68590000 | 180000 | H1-hESC | ESC |
| rs2193049 | AMR | 0.90348 | Within loop | chr12:68400000-68410000 | chr12:68580000-68590000 | 180000 | H1-hESC | ESC |
| rs2193049 | EAS | 0.92889 | Within loop | chr12:68400000-68410000 | chr12:68580000-68590000 | 180000 | H1-hESC | ESC |
| rs2193049 | EUR | 0.96569 | Within loop | chr12:68400000-68410000 | chr12:68580000-68590000 | 180000 | H1-hESC | ESC |
| rs2193049 | SAS | 0.95372 | Within loop | chr12:68400000-68410000 | chr12:68580000-68590000 | 180000 | H1-hESC | ESC |
| rs2193050 | AMR | 0.98633 | Within loop | chr12:68400000-68410000 | chr12:68580000-68590000 | 180000 | H1-hESC | ESC |
| rs2193050 | EAS | 1 | Within loop | chr12:68400000-68410000 | chr12:68580000-68590000 | 180000 | H1-hESC | ESC |
| rs2193050 | EUR | 1 | Within loop | chr12:68400000-68410000 | chr12:68580000-68590000 | 180000 | H1-hESC | ESC |
| rs2193050 | SAS | 0.99209 | Within loop | chr12:68400000-68410000 | chr12:68580000-68590000 | 180000 | H1-hESC | ESC |
| rs2216163 | AMR | 0.98633 | Within loop | chr12:68400000-68410000 | chr12:68580000-68590000 | 180000 | H1-hESC | ESC |
| rs2216163 | EAS | 0.97205 | Within loop | chr12:68400000-68410000 | chr12:68580000-68590000 | 180000 | H1-hESC | ESC |
| rs2216163 | EUR | 0.98494 | Within loop | chr12:68400000-68410000 | chr12:68580000-68590000 | 180000 | H1-hESC | ESC |
| rs2216163 | SAS | 0.9842 | Within loop | chr12:68400000-68410000 | chr12:68580000-68590000 | 180000 | H1-hESC | ESC |
| rs2870952 | AMR | 0.94047 | Within loop | chr12:68400000-68410000 | chr12:68580000-68590000 | 180000 | H1-hESC | ESC |
| rs2870952 | EUR | 0.98003 | Within loop | chr12:68400000-68410000 | chr12:68580000-68590000 | 180000 | H1-hESC | ESC |
| rs2870952 | SAS | 0.96842 | Within loop | chr12:68400000-68410000 | chr12:68580000-68590000 | 180000 | H1-hESC | ESC |
| rs2870953 | AMR | 0.95326 | Within loop | chr12:68400000-68410000 | chr12:68580000-68590000 | 180000 | H1-hESC | ESC |
| rs2870953 | EAS | 1 | Within loop | chr12:68400000-68410000 | chr12:68580000-68590000 | 180000 | H1-hESC | ESC |
| rs2870953 | EUR | 0.99499 | Within loop | chr12:68400000-68410000 | chr12:68580000-68590000 | 180000 | H1-hESC | ESC |
| rs2870953 | SAS | 0.9842 | Within loop | chr12:68400000-68410000 | chr12:68580000-68590000 | 180000 | H1-hESC | ESC |
| rs2904536 | AMR | 1 | Within loop | chr12:68400000-68410000 | chr12:68580000-68590000 | 180000 | H1-hESC | ESC |
| rs2904536 | EUR | 0.98498 | Within loop | chr12:68400000-68410000 | chr12:68580000-68590000 | 180000 | H1-hESC | ESC |
| rs2904536 | SAS | 0.96062 | Within loop | chr12:68400000-68410000 | chr12:68580000-68590000 | 180000 | H1-hESC | ESC |
| rs3181034 | AMR | 0.95326 | Within loop | chr12:68400000-68410000 | chr12:68580000-68590000 | 180000 | H1-hESC | ESC |
| rs3181034 | EAS | 1 | Within loop | chr12:68400000-68410000 | chr12:68580000-68590000 | 180000 | H1-hESC | ESC |
| rs3181034 | EUR | 0.99499 | Within loop | chr12:68400000-68410000 | chr12:68580000-68590000 | 180000 | H1-hESC | ESC |
| rs3181034 | SAS | 0.99209 | Within loop | chr12:68400000-68410000 | chr12:68580000-68590000 | 180000 | H1-hESC | ESC |
| rs4421820 | AMR | 0.92172 | Within loop | chr12:68400000-68410000 | chr12:68580000-68590000 | 180000 | H1-hESC | ESC |
| rs4421820 | EAS | 0.86641 | Within loop | chr12:68400000-68410000 | chr12:68580000-68590000 | 180000 | H1-hESC | ESC |
| rs4421820 | EUR | 0.9606 | Within loop | chr12:68400000-68410000 | chr12:68580000-68590000 | 180000 | H1-hESC | ESC |
| rs4913275 | AMR | 1 | Within loop | chr12:68400000-68410000 | chr12:68580000-68590000 | 180000 | H1-hESC | ESC |
| rs4913275 | EUR | 0.98498 | Within loop | chr12:68400000-68410000 | chr12:68580000-68590000 | 180000 | H1-hESC | ESC |
| rs4913275 | SAS | 0.96842 | Within loop | chr12:68400000-68410000 | chr12:68580000-68590000 | 180000 | H1-hESC | ESC |
| rs4913278 | AMR | 0.93416 | Within loop | chr12:68400000-68410000 | chr12:68580000-68590000 | 180000 | H1-hESC | ESC |
| rs4913278 | EUR | 0.98003 | Within loop | chr12:68400000-68410000 | chr12:68580000-68590000 | 180000 | H1-hESC | ESC |
| rs4913278 | SAS | 0.87482 | Within loop | chr12:68400000-68410000 | chr12:68580000-68590000 | 180000 | H1-hESC | ESC |
| rs4913407 | AMR | 0.9663 | Within loop | chr12:68400000-68410000 | chr12:68580000-68590000 | 180000 | H1-hESC | ESC |
| rs4913407 | EUR | 0.98498 | Within loop | chr12:68400000-68410000 | chr12:68580000-68590000 | 180000 | H1-hESC | ESC |
| rs4913407 | SAS | 0.94488 | Within loop | chr12:68400000-68410000 | chr12:68580000-68590000 | 180000 | H1-hESC | ESC |
| rs4913408 | AMR | 1 | Within loop | chr12:68400000-68410000 | chr12:68580000-68590000 | 180000 | H1-hESC | ESC |
| rs4913408 | EUR | 0.97995 | Within loop | chr12:68400000-68410000 | chr12:68580000-68590000 | 180000 | H1-hESC | ESC |
| rs4913408 | SAS | 0.94488 | Within loop | chr12:68400000-68410000 | chr12:68580000-68590000 | 180000 | H1-hESC | ESC |
| rs4913411 | AMR | 0.9663 | Within loop | chr12:68400000-68410000 | chr12:68580000-68590000 | 180000 | H1-hESC | ESC |
| rs4913411 | EUR | 0.98498 | Within loop | chr12:68400000-68410000 | chr12:68580000-68590000 | 180000 | H1-hESC | ESC |
| rs4913411 | SAS | 0.96842 | Within loop | chr12:68400000-68410000 | chr12:68580000-68590000 | 180000 | H1-hESC | ESC |
| rs4913412 | AMR | 0.9663 | Within loop | chr12:68400000-68410000 | chr12:68580000-68590000 | 180000 | H1-hESC | ESC |
| rs4913412 | EUR | 0.98498 | Within loop | chr12:68400000-68410000 | chr12:68580000-68590000 | 180000 | H1-hESC | ESC |
| rs4913412 | SAS | 0.96842 | Within loop | chr12:68400000-68410000 | chr12:68580000-68590000 | 180000 | H1-hESC | ESC |
| rs4913415 | AMR | 0.95975 | Within loop | chr12:68400000-68410000 | chr12:68580000-68590000 | 180000 | H1-hESC | ESC |
| rs4913415 | EUR | 0.97512 | Within loop | chr12:68400000-68410000 | chr12:68580000-68590000 | 180000 | H1-hESC | ESC |
| rs4913415 | SAS | 0.86711 | Within loop | chr12:68400000-68410000 | chr12:68580000-68590000 | 180000 | H1-hESC | ESC |
| rs55883501 | AMR | 0.94047 | Within loop | chr12:68400000-68410000 | chr12:68580000-68590000 | 180000 | H1-hESC | ESC |
| rs55883501 | EUR | 0.98003 | Within loop | chr12:68400000-68410000 | chr12:68580000-68590000 | 180000 | H1-hESC | ESC |
| rs55883501 | SAS | 0.95292 | Within loop | chr12:68400000-68410000 | chr12:68580000-68590000 | 180000 | H1-hESC | ESC |
| rs57781022 | AMR | 0.96573 | Within loop | chr12:68400000-68410000 | chr12:68580000-68590000 | 180000 | H1-hESC | ESC |
| rs57781022 | EAS | 0.977 | Within loop | chr12:68400000-68410000 | chr12:68580000-68590000 | 180000 | H1-hESC | ESC |
| rs57781022 | EUR | 0.96093 | Within loop | chr12:68400000-68410000 | chr12:68580000-68590000 | 180000 | H1-hESC | ESC |
| rs57781022 | SAS | 0.93188 | Within loop | chr12:68400000-68410000 | chr12:68580000-68590000 | 180000 | H1-hESC | ESC |
| rs6421226 | AMR | 0.9663 | Within loop | chr12:68400000-68410000 | chr12:68580000-68590000 | 180000 | H1-hESC | ESC |
| rs6421226 | EUR | 0.98498 | Within loop | chr12:68400000-68410000 | chr12:68580000-68590000 | 180000 | H1-hESC | ESC |
| rs6421226 | SAS | 0.96842 | Within loop | chr12:68400000-68410000 | chr12:68580000-68590000 | 180000 | H1-hESC | ESC |
| rs6421227 | AMR | 1 | Within loop | chr12:68400000-68410000 | chr12:68580000-68590000 | 180000 | H1-hESC | ESC |
| rs6421227 | EUR | 0.98498 | Within loop | chr12:68400000-68410000 | chr12:68580000-68590000 | 180000 | H1-hESC | ESC |
| rs6421227 | SAS | 0.96842 | Within loop | chr12:68400000-68410000 | chr12:68580000-68590000 | 180000 | H1-hESC | ESC |
| rs6581790 | AMR | 0.98633 | Within loop | chr12:68400000-68410000 | chr12:68580000-68590000 | 180000 | H1-hESC | ESC |
| rs6581790 | EAS | 0.97205 | Within loop | chr12:68400000-68410000 | chr12:68580000-68590000 | 180000 | H1-hESC | ESC |
| rs6581790 | EUR | 0.98997 | Within loop | chr12:68400000-68410000 | chr12:68580000-68590000 | 180000 | H1-hESC | ESC |
| rs6581790 | SAS | 0.9842 | Within loop | chr12:68400000-68410000 | chr12:68580000-68590000 | 180000 | H1-hESC | ESC |
| rs6581792 | AMR | 0.98633 | Within loop | chr12:68400000-68410000 | chr12:68580000-68590000 | 180000 | H1-hESC | ESC |
| rs6581792 | EAS | 1 | Within loop | chr12:68400000-68410000 | chr12:68580000-68590000 | 180000 | H1-hESC | ESC |
| rs6581792 | EUR | 1 | Within loop | chr12:68400000-68410000 | chr12:68580000-68590000 | 180000 | H1-hESC | ESC |
| rs6581792 | SAS | 0.97633 | Within loop | chr12:68400000-68410000 | chr12:68580000-68590000 | 180000 | H1-hESC | ESC |
| rs6581793 | AMR | 0.92172 | Within loop | chr12:68400000-68410000 | chr12:68580000-68590000 | 180000 | H1-hESC | ESC |
| rs6581793 | EAS | 1 | Within loop | chr12:68400000-68410000 | chr12:68580000-68590000 | 180000 | H1-hESC | ESC |
| rs6581793 | EUR | 0.99002 | Within loop | chr12:68400000-68410000 | chr12:68580000-68590000 | 180000 | H1-hESC | ESC |
| rs6581793 | SAS | 0.9842 | Within loop | chr12:68400000-68410000 | chr12:68580000-68590000 | 180000 | H1-hESC | ESC |
| rs6581794 | AMR | 0.92172 | Within loop | chr12:68400000-68410000 | chr12:68580000-68590000 | 180000 | H1-hESC | ESC |
| rs6581794 | EAS | 1 | Within loop | chr12:68400000-68410000 | chr12:68580000-68590000 | 180000 | H1-hESC | ESC |
| rs6581794 | EUR | 0.99499 | Within loop | chr12:68400000-68410000 | chr12:68580000-68590000 | 180000 | H1-hESC | ESC |
| rs6581794 | SAS | 0.9842 | Within loop | chr12:68400000-68410000 | chr12:68580000-68590000 | 180000 | H1-hESC | ESC |
| rs6581795 | AMR | 1 | Within loop | chr12:68400000-68410000 | chr12:68580000-68590000 | 180000 | H1-hESC | ESC |
| rs6581795 | EUR | 0.98498 | Within loop | chr12:68400000-68410000 | chr12:68580000-68590000 | 180000 | H1-hESC | ESC |
| rs6581795 | SAS | 0.96842 | Within loop | chr12:68400000-68410000 | chr12:68580000-68590000 | 180000 | H1-hESC | ESC |
| rs7132697 | AMR | 0.98633 | Within loop | chr12:68400000-68410000 | chr12:68580000-68590000 | 180000 | H1-hESC | ESC |
| rs7132697 | EAS | 0.98603 | Within loop | chr12:68400000-68410000 | chr12:68580000-68590000 | 180000 | H1-hESC | ESC |
| rs7132697 | EUR | 0.98494 | Within loop | chr12:68400000-68410000 | chr12:68580000-68590000 | 180000 | H1-hESC | ESC |
| rs7132697 | SAS | 0.9842 | Within loop | chr12:68400000-68410000 | chr12:68580000-68590000 | 180000 | H1-hESC | ESC |
| rs7133554 | AMR | 0.98633 | Within loop | chr12:68400000-68410000 | chr12:68580000-68590000 | 180000 | H1-hESC | ESC |
| rs7133554 | EAS | 0.97205 | Within loop | chr12:68400000-68410000 | chr12:68580000-68590000 | 180000 | H1-hESC | ESC |
| rs7133554 | EUR | 0.98997 | Within loop | chr12:68400000-68410000 | chr12:68580000-68590000 | 180000 | H1-hESC | ESC |
| rs7133554 | SAS | 0.9842 | Within loop | chr12:68400000-68410000 | chr12:68580000-68590000 | 180000 | H1-hESC | ESC |
| rs7133709 | AMR | 0.9663 | Within loop | chr12:68400000-68410000 | chr12:68580000-68590000 | 180000 | H1-hESC | ESC |
| rs7133709 | EUR | 0.98498 | Within loop | chr12:68400000-68410000 | chr12:68580000-68590000 | 180000 | H1-hESC | ESC |
| rs7133709 | SAS | 0.96842 | Within loop | chr12:68400000-68410000 | chr12:68580000-68590000 | 180000 | H1-hESC | ESC |
| rs7135373 | AMR | 0.9663 | Within loop | chr12:68400000-68410000 | chr12:68580000-68590000 | 180000 | H1-hESC | ESC |
| rs7135373 | EUR | 0.98498 | Within loop | chr12:68400000-68410000 | chr12:68580000-68590000 | 180000 | H1-hESC | ESC |
| rs7135373 | SAS | 0.96062 | Within loop | chr12:68400000-68410000 | chr12:68580000-68590000 | 180000 | H1-hESC | ESC |
| rs7138107 | AMR | 0.9663 | Within loop | chr12:68400000-68410000 | chr12:68580000-68590000 | 180000 | H1-hESC | ESC |
| rs7138107 | EUR | 0.98003 | Within loop | chr12:68400000-68410000 | chr12:68580000-68590000 | 180000 | H1-hESC | ESC |
| rs7138107 | SAS | 0.96842 | Within loop | chr12:68400000-68410000 | chr12:68580000-68590000 | 180000 | H1-hESC | ESC |
| rs7139169 | AMR | 0.9095 | Within loop | chr12:68400000-68410000 | chr12:68580000-68590000 | 180000 | H1-hESC | ESC |
| rs7139169 | EUR | 0.99002 | Within loop | chr12:68400000-68410000 | chr12:68580000-68590000 | 180000 | H1-hESC | ESC |
| rs7139169 | SAS | 0.96842 | Within loop | chr12:68400000-68410000 | chr12:68580000-68590000 | 180000 | H1-hESC | ESC |
| rs72648183 | EAS | 0.9767 | Within loop | chr12:68400000-68410000 | chr12:68580000-68590000 | 180000 | H1-hESC | ESC |
| rs7302226 | AMR | 0.98633 | Within loop | chr12:68400000-68410000 | chr12:68580000-68590000 | 180000 | H1-hESC | ESC |
| rs7302226 | EAS | 0.97205 | Within loop | chr12:68400000-68410000 | chr12:68580000-68590000 | 180000 | H1-hESC | ESC |
| rs7302226 | EUR | 0.98997 | Within loop | chr12:68400000-68410000 | chr12:68580000-68590000 | 180000 | H1-hESC | ESC |
| rs7302226 | SAS | 0.9842 | Within loop | chr12:68400000-68410000 | chr12:68580000-68590000 | 180000 | H1-hESC | ESC |
| rs7302488 | AMR | 0.94684 | Within loop | chr12:68400000-68410000 | chr12:68580000-68590000 | 180000 | H1-hESC | ESC |
| rs7302488 | EUR | 0.98498 | Within loop | chr12:68400000-68410000 | chr12:68580000-68590000 | 180000 | H1-hESC | ESC |
| rs7302488 | SAS | 0.96842 | Within loop | chr12:68400000-68410000 | chr12:68580000-68590000 | 180000 | H1-hESC | ESC |
| rs7306196 | AMR | 0.95932 | Within loop | chr12:68400000-68410000 | chr12:68580000-68590000 | 180000 | H1-hESC | ESC |
| rs7306196 | EUR | 0.98498 | Within loop | chr12:68400000-68410000 | chr12:68580000-68590000 | 180000 | H1-hESC | ESC |
| rs7306196 | SAS | 0.96842 | Within loop | chr12:68400000-68410000 | chr12:68580000-68590000 | 180000 | H1-hESC | ESC |
| rs759487 | AMR | 1 | Within loop | chr12:68400000-68410000 | chr12:68580000-68590000 | 180000 | H1-hESC | ESC |
| rs759487 | EUR | 0.98498 | Within loop | chr12:68400000-68410000 | chr12:68580000-68590000 | 180000 | H1-hESC | ESC |
| rs759487 | SAS | 0.96842 | Within loop | chr12:68400000-68410000 | chr12:68580000-68590000 | 180000 | H1-hESC | ESC |
| rs759488 | EUR | 0.97025 | Within loop | chr12:68400000-68410000 | chr12:68580000-68590000 | 180000 | H1-hESC | ESC |
| rs759488 | SAS | 0.83686 | Within loop | chr12:68400000-68410000 | chr12:68580000-68590000 | 180000 | H1-hESC | ESC |
| rs7959933 | AMR | 0.93416 | Within loop | chr12:68400000-68410000 | chr12:68580000-68590000 | 180000 | H1-hESC | ESC |
| rs7959933 | EUR | 0.98003 | Within loop | chr12:68400000-68410000 | chr12:68580000-68590000 | 180000 | H1-hESC | ESC |
| rs7959933 | SAS | 0.87482 | Within loop | chr12:68400000-68410000 | chr12:68580000-68590000 | 180000 | H1-hESC | ESC |
| rs7969745 | AMR | 0.92172 | Within loop | chr12:68400000-68410000 | chr12:68580000-68590000 | 180000 | H1-hESC | ESC |
| rs7969745 | EAS | 0.97671 | Within loop | chr12:68400000-68410000 | chr12:68580000-68590000 | 180000 | H1-hESC | ESC |
| rs7969745 | EUR | 0.98495 | Within loop | chr12:68400000-68410000 | chr12:68580000-68590000 | 180000 | H1-hESC | ESC |
| rs7969745 | SAS | 0.9842 | Within loop | chr12:68400000-68410000 | chr12:68580000-68590000 | 180000 | H1-hESC | ESC |
| rs7973170 | AMR | 0.98633 | Within loop | chr12:68400000-68410000 | chr12:68580000-68590000 | 180000 | H1-hESC | ESC |
| rs7973170 | EAS | 0.98603 | Within loop | chr12:68400000-68410000 | chr12:68580000-68590000 | 180000 | H1-hESC | ESC |
| rs7973170 | EUR | 0.98494 | Within loop | chr12:68400000-68410000 | chr12:68580000-68590000 | 180000 | H1-hESC | ESC |
| rs7973170 | SAS | 0.97633 | Within loop | chr12:68400000-68410000 | chr12:68580000-68590000 | 180000 | H1-hESC | ESC |
| rs7978128 | AMR | 0.98633 | Within loop | chr12:68400000-68410000 | chr12:68580000-68590000 | 180000 | H1-hESC | ESC |
| rs7978128 | EAS | 0.99533 | Within loop | chr12:68400000-68410000 | chr12:68580000-68590000 | 180000 | H1-hESC | ESC |
| rs7978128 | EUR | 1 | Within loop | chr12:68400000-68410000 | chr12:68580000-68590000 | 180000 | H1-hESC | ESC |
| rs7978128 | SAS | 0.9842 | Within loop | chr12:68400000-68410000 | chr12:68580000-68590000 | 180000 | H1-hESC | ESC |
| rs9668812 | AMR | 0.98633 | Within loop | chr12:68400000-68410000 | chr12:68580000-68590000 | 180000 | H1-hESC | ESC |
| rs9668812 | EAS | 0.99533 | Within loop | chr12:68400000-68410000 | chr12:68580000-68590000 | 180000 | H1-hESC | ESC |
| rs9668812 | EUR | 1 | Within loop | chr12:68400000-68410000 | chr12:68580000-68590000 | 180000 | H1-hESC | ESC |
| rs9668812 | SAS | 0.9842 | Within loop | chr12:68400000-68410000 | chr12:68580000-68590000 | 180000 | H1-hESC | ESC |
| rs9888400 | AMR | 0.94047 | Within loop | chr12:68400000-68410000 | chr12:68580000-68590000 | 180000 | H1-hESC | ESC |
| rs9888400 | EUR | 0.97512 | Within loop | chr12:68400000-68410000 | chr12:68580000-68590000 | 180000 | H1-hESC | ESC |
| rs9888400 | SAS | 0.92929 | Within loop | chr12:68400000-68410000 | chr12:68580000-68590000 | 180000 | H1-hESC | ESC |

African: AFR, Ad Mixed American: AMR, East Asian: ASN, European: EUR, and South Asian: SAS.

| **Supplemental Table 8. The rs2069718 associated and interacted single nucleotide polymorphisms (SNPs) through three-dimensional (3D) chromatin looping (LD r^2^ > 0.8).** | | | | | | | | |
| --- | --- | --- | --- | --- | --- | --- | --- | --- |
| **SNP ID** | **Population** | **LD(r2)** | **Loop type** | **Loop start** | **Loop end** | **Distance** | **Cell type** | **Tissue** |
| rs1076025 | AMR | 0.8509 | Within loop | chr12:68400000-68410000 | chr12:68580000-68590000 | 180000 | H1-hESC | ESC |
| rs1076025 | EUR | 0.90684 | Within loop | chr12:68400000-68410000 | chr12:68580000-68590000 | 180000 | H1-hESC | ESC |
| rs10878756 | AMR | 0.88923 | Within loop | chr12:68400000-68410000 | chr12:68580000-68590000 | 180000 | H1-hESC | ESC |
| rs10878756 | EAS | 0.92873 | Within loop | chr12:68400000-68410000 | chr12:68580000-68590000 | 180000 | H1-hESC | ESC |
| rs10878756 | EUR | 0.8987 | Within loop | chr12:68400000-68410000 | chr12:68580000-68590000 | 180000 | H1-hESC | ESC |
| rs12312186 | AMR | 0.8355 | Within loop | chr12:68400000-68410000 | chr12:68580000-68590000 | 180000 | H1-hESC | ESC |
| rs12312186 | EUR | 0.90684 | Within loop | chr12:68400000-68410000 | chr12:68580000-68590000 | 180000 | H1-hESC | ESC |
| rs12313214 | AMR | 0.98122 | Within loop | chr12:68400000-68410000 | chr12:68580000-68590000 | 180000 | H1-hESC | ESC |
| rs12313214 | EAS | 0.9355 | Within loop | chr12:68400000-68410000 | chr12:68580000-68590000 | 180000 | H1-hESC | ESC |
| rs12313214 | EUR | 0.97523 | Within loop | chr12:68400000-68410000 | chr12:68580000-68590000 | 180000 | H1-hESC | ESC |
| rs12313214 | SAS | 0.97111 | Within loop | chr12:68400000-68410000 | chr12:68580000-68590000 | 180000 | H1-hESC | ESC |
| rs12315837 | AMR | 0.83044 | Within loop | chr12:68400000-68410000 | chr12:68580000-68590000 | 180000 | H1-hESC | ESC |
| rs12315837 | EUR | 0.90684 | Within loop | chr12:68400000-68410000 | chr12:68580000-68590000 | 180000 | H1-hESC | ESC |
| rs2041864 | AMR | 0.98751 | Within loop | chr12:68400000-68410000 | chr12:68580000-68590000 | 180000 | H1-hESC | ESC |
| rs2041864 | EAS | 0.98526 | Within loop | chr12:68400000-68410000 | chr12:68580000-68590000 | 180000 | H1-hESC | ESC |
| rs2041864 | EUR | 0.98757 | Within loop | chr12:68400000-68410000 | chr12:68580000-68590000 | 180000 | H1-hESC | ESC |
| rs2041864 | SAS | 0.99586 | Within loop | chr12:68400000-68410000 | chr12:68580000-68590000 | 180000 | H1-hESC | ESC |
| rs2058739 | EUR | 0.90234 | Within loop | chr12:68400000-68410000 | chr12:68580000-68590000 | 180000 | H1-hESC | ESC |
| rs2069727 | EAS | 0.98526 | Within loop | chr12:68400000-68410000 | chr12:68580000-68590000 | 180000 | H1-hESC | ESC |
| rs2069727 | SAS | 0.8102 | Within loop | chr12:68400000-68410000 | chr12:68580000-68590000 | 180000 | H1-hESC | ESC |
| rs2080414 | AMR | 0.83044 | Within loop | chr12:68400000-68410000 | chr12:68580000-68590000 | 180000 | H1-hESC | ESC |
| rs2080414 | EUR | 0.90684 | Within loop | chr12:68400000-68410000 | chr12:68580000-68590000 | 180000 | H1-hESC | ESC |
| rs2193046 | EAS | 0.97052 | Within loop | chr12:68400000-68410000 | chr12:68580000-68590000 | 180000 | H1-hESC | ESC |
| rs2216164 | AMR | 0.98751 | Within loop | chr12:68400000-68410000 | chr12:68580000-68590000 | 180000 | H1-hESC | ESC |
| rs2216164 | EAS | 0.97788 | Within loop | chr12:68400000-68410000 | chr12:68580000-68590000 | 180000 | H1-hESC | ESC |
| rs2216164 | EUR | 0.98757 | Within loop | chr12:68400000-68410000 | chr12:68580000-68590000 | 180000 | H1-hESC | ESC |
| rs2216164 | SAS | 0.9876 | Within loop | chr12:68400000-68410000 | chr12:68580000-68590000 | 180000 | H1-hESC | ESC |
| rs2430561 | EAS | 0.99262 | Within loop | chr12:68400000-68410000 | chr12:68580000-68590000 | 180000 | H1-hESC | ESC |
| rs2430561 | SAS | 0.80327 | Within loop | chr12:68400000-68410000 | chr12:68580000-68590000 | 180000 | H1-hESC | ESC |
| rs2870951 | AMR | 0.83463 | Within loop | chr12:68400000-68410000 | chr12:68580000-68590000 | 180000 | H1-hESC | ESC |
| rs2870951 | EUR | 0.94233 | Within loop | chr12:68400000-68410000 | chr12:68580000-68590000 | 180000 | H1-hESC | ESC |
| rs35314021 | EAS | 0.84371 | Within loop | chr12:68400000-68410000 | chr12:68580000-68590000 | 180000 | H1-hESC | ESC |
| rs4913277 | EUR | 0.89816 | Within loop | chr12:68400000-68410000 | chr12:68580000-68590000 | 180000 | H1-hESC | ESC |
| rs4913416 | EUR | 0.90234 | Within loop | chr12:68400000-68410000 | chr12:68580000-68590000 | 180000 | H1-hESC | ESC |
| rs7137814 | AMR | 0.8509 | Within loop | chr12:68400000-68410000 | chr12:68580000-68590000 | 180000 | H1-hESC | ESC |
| rs7137814 | EUR | 0.90684 | Within loop | chr12:68400000-68410000 | chr12:68580000-68590000 | 180000 | H1-hESC | ESC |
| rs7137993 | AMR | 0.8509 | Within loop | chr12:68400000-68410000 | chr12:68580000-68590000 | 180000 | H1-hESC | ESC |
| rs7137993 | EUR | 0.90684 | Within loop | chr12:68400000-68410000 | chr12:68580000-68590000 | 180000 | H1-hESC | ESC |
| rs7297259 | EUR | 0.88566 | Within loop | chr12:68400000-68410000 | chr12:68580000-68590000 | 180000 | H1-hESC | ESC |
| rs7298410 | EUR | 0.90234 | Within loop | chr12:68400000-68410000 | chr12:68580000-68590000 | 180000 | H1-hESC | ESC |
| rs7304531 | AMR | 0.98122 | Within loop | chr12:68400000-68410000 | chr12:68580000-68590000 | 180000 | H1-hESC | ESC |
| rs7304531 | EAS | 0.9355 | Within loop | chr12:68400000-68410000 | chr12:68580000-68590000 | 180000 | H1-hESC | ESC |
| rs7304531 | EUR | 0.97932 | Within loop | chr12:68400000-68410000 | chr12:68580000-68590000 | 180000 | H1-hESC | ESC |
| rs7304531 | SAS | 0.96296 | Within loop | chr12:68400000-68410000 | chr12:68580000-68590000 | 180000 | H1-hESC | ESC |
| rs7954499 | EAS | 0.99262 | Within loop | chr12:68400000-68410000 | chr12:68580000-68590000 | 180000 | H1-hESC | ESC |
| rs7954499 | SAS | 0.8102 | Within loop | chr12:68400000-68410000 | chr12:68580000-68590000 | 180000 | H1-hESC | ESC |
| rs7956817 | AMR | 0.83044 | Within loop | chr12:68400000-68410000 | chr12:68580000-68590000 | 180000 | H1-hESC | ESC |
| rs7956817 | EUR | 0.90684 | Within loop | chr12:68400000-68410000 | chr12:68580000-68590000 | 180000 | H1-hESC | ESC |
| rs7968411 | AMR | 0.88895 | Within loop | chr12:68400000-68410000 | chr12:68580000-68590000 | 180000 | H1-hESC | ESC |
| rs7968411 | EUR | 0.95055 | Within loop | chr12:68400000-68410000 | chr12:68580000-68590000 | 180000 | H1-hESC | ESC |
| rs7968411 | SAS | 0.90992 | Within loop | chr12:68400000-68410000 | chr12:68580000-68590000 | 180000 | H1-hESC | ESC |
| rs9888319 | AMR | 0.82382 | Within loop | chr12:68400000-68410000 | chr12:68580000-68590000 | 180000 | H1-hESC | ESC |
| rs9888319 | EUR | 0.90234 | Within loop | chr12:68400000-68410000 | chr12:68580000-68590000 | 180000 | H1-hESC | ESC |
| rs9888402 | AMR | 0.82382 | Within loop | chr12:68400000-68410000 | chr12:68580000-68590000 | 180000 | H1-hESC | ESC |
| rs9888402 | EUR | 0.90234 | Within loop | chr12:68400000-68410000 | chr12:68580000-68590000 | 180000 | H1-hESC | ESC |

African: AFR, Ad Mixed American: AMR, East Asian: ASN, European: EUR, and South Asian: SAS.

| **Supplemental Table 9. The rs9376267 associated and interacted single nucleotide polymorphisms (SNPs) through three-dimensional (3D) chromatin looping (LD r^2^ > 0.8).** | | | | | | | | |
| --- | --- | --- | --- | --- | --- | --- | --- | --- |
| **SNP ID** | **Population** | **LD(r2)** | **Loop type** | **Loop start** | **Loop end** | **Distance** | **Cell type** | **Tissue** |
| rs150776768 | AMR | 0.84585 | Within loop | chr6:137420000-137425000 | chr6:137535000-137540000 | 115000 | GM12878 | Blood |
| rs150776768 | EUR | 0.94843 | Within loop | chr6:137420000-137425000 | chr6:137535000-137540000 | 115000 | GM12878 | Blood |
| rs376706088 | EUR | 0.91313 | Within loop | chr6:137420000-137425000 | chr6:137535000-137540000 | 115000 | GM12878 | Blood |
| rs542331996 | AMR | 0.81405 | Within loop | chr6:137420000-137425000 | chr6:137535000-137540000 | 115000 | GM12878 | Blood |
| rs542331996 | EUR | 0.9172 | Within loop | chr6:137420000-137425000 | chr6:137535000-137540000 | 115000 | GM12878 | Blood |
| rs56251346 | AMR | 0.85417 | Within loop | chr6:137420000-137425000 | chr6:137535000-137540000 | 115000 | GM12878 | Blood |
| rs56251346 | EUR | 0.95352 | Within loop | chr6:137420000-137425000 | chr6:137535000-137540000 | 115000 | GM12878 | Blood |
| rs75537328 | AMR | 0.90072 | Within loop | chr6:137420000-137425000 | chr6:137535000-137540000 | 115000 | GM12878 | Blood |
| rs75537328 | EAS | 0.89969 | Within loop | chr6:137420000-137425000 | chr6:137535000-137540000 | 115000 | GM12878 | Blood |
| rs75537328 | EUR | 0.93 | Within loop | chr6:137420000-137425000 | chr6:137535000-137540000 | 115000 | GM12878 | Blood |
| rs75537328 | SAS | 0.93012 | Within loop | chr6:137420000-137425000 | chr6:137535000-137540000 | 115000 | GM12878 | Blood |
| rs7759040 | AMR | 0.96857 | Within loop | chr6:137420000-137425000 | chr6:137535000-137540000 | 115000 | GM12878 | Blood |
| rs7759040 | EAS | 0.94902 | Within loop | chr6:137420000-137425000 | chr6:137535000-137540000 | 115000 | GM12878 | Blood |
| rs7759040 | EUR | 0.99478 | Within loop | chr6:137420000-137425000 | chr6:137535000-137540000 | 115000 | GM12878 | Blood |
| rs7759040 | SAS | 1 | Within loop | chr6:137420000-137425000 | chr6:137535000-137540000 | 115000 | GM12878 | Blood |
| rs9376268 | AMR | 0.85417 | Within loop | chr6:137420000-137425000 | chr6:137535000-137540000 | 115000 | GM12878 | Blood |
| rs9376268 | EUR | 0.95352 | Within loop | chr6:137420000-137425000 | chr6:137535000-137540000 | 115000 | GM12878 | Blood |
| rs9376269 | AMR | 0.98414 | Within loop | chr6:137420000-137425000 | chr6:137535000-137540000 | 115000 | GM12878 | Blood |
| rs9376269 | EAS | 0.95307 | Within loop | chr6:137420000-137425000 | chr6:137535000-137540000 | 115000 | GM12878 | Blood |
| rs9376269 | EUR | 0.98435 | Within loop | chr6:137420000-137425000 | chr6:137535000-137540000 | 115000 | GM12878 | Blood |
| rs9376269 | SAS | 0.99171 | Within loop | chr6:137420000-137425000 | chr6:137535000-137540000 | 115000 | GM12878 | Blood |
| rs9389480 | AMR | 0.85417 | Within loop | chr6:137420000-137425000 | chr6:137535000-137540000 | 115000 | GM12878 | Blood |
| rs9389480 | EUR | 0.95352 | Within loop | chr6:137420000-137425000 | chr6:137535000-137540000 | 115000 | GM12878 | Blood |
| rs9389484 | AMR | 0.85417 | Within loop | chr6:137420000-137425000 | chr6:137535000-137540000 | 115000 | GM12878 | Blood |
| rs9389484 | EUR | 0.95352 | Within loop | chr6:137420000-137425000 | chr6:137535000-137540000 | 115000 | GM12878 | Blood |
| rs9402879 | AMR | 0.85417 | Within loop | chr6:137420000-137425000 | chr6:137535000-137540000 | 115000 | GM12878 | Blood |
| rs9402879 | EUR | 0.95863 | Within loop | chr6:137420000-137425000 | chr6:137535000-137540000 | 115000 | GM12878 | Blood |

African: AFR, Ad Mixed American: AMR, East Asian: ASN, European: EUR, and South Asian: SAS.

| **Supplemental Table 10. The rs9376268 associated and interacted single nucleotide polymorphisms (SNPs) through three-dimensional (3D) chromatin looping (LD r^2^ > 0.8).** | | | | | | | | |
| --- | --- | --- | --- | --- | --- | --- | --- | --- |
| **SNP ID** | **Population** | **LD(r2)** | **Loop type** | **Loop start** | **Loop end** | **Distance** | **Cell type** | **Tissue** |
| rs150776768 | AMR | 0.99139 | Within loop | chr6:137420000-137425000 | chr6:137535000-137540000 | 115000 | GM12878 | Blood |
| rs150776768 | EAS | 0.80795 | Within loop | chr6:137420000-137425000 | chr6:137535000-137540000 | 115000 | GM12878 | Blood |
| rs150776768 | EUR | 0.99466 | Within loop | chr6:137420000-137425000 | chr6:137535000-137540000 | 115000 | GM12878 | Blood |
| rs150776768 | SAS | 0.99459 | Within loop | chr6:137420000-137425000 | chr6:137535000-137540000 | 115000 | GM12878 | Blood |
| rs376706088 | AMR | 0.91406 | Within loop | chr6:137420000-137425000 | chr6:137535000-137540000 | 115000 | GM12878 | Blood |
| rs376706088 | EAS | 0.97538 | Within loop | chr6:137420000-137425000 | chr6:137535000-137540000 | 115000 | GM12878 | Blood |
| rs376706088 | EUR | 0.94698 | Within loop | chr6:137420000-137425000 | chr6:137535000-137540000 | 115000 | GM12878 | Blood |
| rs376706088 | SAS | 0.96763 | Within loop | chr6:137420000-137425000 | chr6:137535000-137540000 | 115000 | GM12878 | Blood |
| rs542331996 | AMR | 0.95748 | Within loop | chr6:137420000-137425000 | chr6:137535000-137540000 | 115000 | GM12878 | Blood |
| rs542331996 | EAS | 0.95521 | Within loop | chr6:137420000-137425000 | chr6:137535000-137540000 | 115000 | GM12878 | Blood |
| rs542331996 | EUR | 0.95249 | Within loop | chr6:137420000-137425000 | chr6:137535000-137540000 | 115000 | GM12878 | Blood |
| rs542331996 | SAS | 0.92564 | Within loop | chr6:137420000-137425000 | chr6:137535000-137540000 | 115000 | GM12878 | Blood |
| rs56251346 | AMR | 1 | Within loop | chr6:137420000-137425000 | chr6:137535000-137540000 | 115000 | GM12878 | Blood |
| rs56251346 | EAS | 0.96769 | Within loop | chr6:137420000-137425000 | chr6:137535000-137540000 | 115000 | GM12878 | Blood |
| rs56251346 | EUR | 1 | Within loop | chr6:137420000-137425000 | chr6:137535000-137540000 | 115000 | GM12878 | Blood |
| rs56251346 | SAS | 0.9892 | Within loop | chr6:137420000-137425000 | chr6:137535000-137540000 | 115000 | GM12878 | Blood |
| rs75537328 | AMR | 0.80856 | Within loop | chr6:137420000-137425000 | chr6:137535000-137540000 | 115000 | GM12878 | Blood |
| rs75537328 | EUR | 0.89639 | Within loop | chr6:137420000-137425000 | chr6:137535000-137540000 | 115000 | GM12878 | Blood |
| rs7759040 | AMR | 0.84075 | Within loop | chr6:137420000-137425000 | chr6:137535000-137540000 | 115000 | GM12878 | Blood |
| rs7759040 | EUR | 0.95853 | Within loop | chr6:137420000-137425000 | chr6:137535000-137540000 | 115000 | GM12878 | Blood |
| rs9376267 | AMR | 0.85417 | Within loop | chr6:137420000-137425000 | chr6:137535000-137540000 | 115000 | GM12878 | Blood |
| rs9376267 | EUR | 0.95352 | Within loop | chr6:137420000-137425000 | chr6:137535000-137540000 | 115000 | GM12878 | Blood |
| rs9376269 | AMR | 0.83843 | Within loop | chr6:137420000-137425000 | chr6:137535000-137540000 | 115000 | GM12878 | Blood |
| rs9376269 | EUR | 0.9481 | Within loop | chr6:137420000-137425000 | chr6:137535000-137540000 | 115000 | GM12878 | Blood |
| rs9389480 | AMR | 1 | Within loop | chr6:137420000-137425000 | chr6:137535000-137540000 | 115000 | GM12878 | Blood |
| rs9389480 | EAS | 0.96352 | Within loop | chr6:137420000-137425000 | chr6:137535000-137540000 | 115000 | GM12878 | Blood |
| rs9389480 | EUR | 1 | Within loop | chr6:137420000-137425000 | chr6:137535000-137540000 | 115000 | GM12878 | Blood |
| rs9389480 | SAS | 0.9892 | Within loop | chr6:137420000-137425000 | chr6:137535000-137540000 | 115000 | GM12878 | Blood |
| rs9389484 | AMR | 1 | Within loop | chr6:137420000-137425000 | chr6:137535000-137540000 | 115000 | GM12878 | Blood |
| rs9389484 | EAS | 0.9795 | Within loop | chr6:137420000-137425000 | chr6:137535000-137540000 | 115000 | GM12878 | Blood |
| rs9389484 | EUR | 1 | Within loop | chr6:137420000-137425000 | chr6:137535000-137540000 | 115000 | GM12878 | Blood |
| rs9389484 | SAS | 0.99459 | Within loop | chr6:137420000-137425000 | chr6:137535000-137540000 | 115000 | GM12878 | Blood |
| rs9402879 | AMR | 1 | Within loop | chr6:137420000-137425000 | chr6:137535000-137540000 | 115000 | GM12878 | Blood |
| rs9402879 | EAS | 0.9795 | Within loop | chr6:137420000-137425000 | chr6:137535000-137540000 | 115000 | GM12878 | Blood |
| rs9402879 | EUR | 0.99467 | Within loop | chr6:137420000-137425000 | chr6:137535000-137540000 | 115000 | GM12878 | Blood |
| rs9402879 | SAS | 0.98919 | Within loop | chr6:137420000-137425000 | chr6:137535000-137540000 | 115000 | GM12878 | Blood |

African: AFR, Ad Mixed American: AMR, East Asian: ASN, European: EUR, and South Asian: SAS.

| **Supplemental Table 11. The rs10457655 associated and interacted single nucleotide polymorphisms (SNPs) through three-dimensional (3D) chromatin looping (LD r^2^ > 0.8).** | | | | | | | | |
| --- | --- | --- | --- | --- | --- | --- | --- | --- |
| **SNP ID** | **Population** | **LD(r2)** | **Loop type** | **Loop start** | **Loop end** | **Distance** | **Cell type** | **Tissue** |
| rs11914 | EAS | 0.9313 | Within loop | chr6:137420000-137425000 | chr6:137535000-137540000 | 115000 | GM12878 | Blood |
| rs11914 | EUR | 0.96168 | Within loop | chr6:137420000-137425000 | chr6:137535000-137540000 | 115000 | GM12878 | Blood |
| rs11914 | SAS | 0.95785 | Within loop | chr6:137420000-137425000 | chr6:137535000-137540000 | 115000 | GM12878 | Blood |
| rs1327475 | EAS | 0.9313 | Within loop | chr6:137420000-137425000 | chr6:137535000-137540000 | 115000 | GM12878 | Blood |
| rs1327475 | EUR | 0.95407 | Within loop | chr6:137420000-137425000 | chr6:137535000-137540000 | 115000 | GM12878 | Blood |
| rs1327475 | SAS | 0.95785 | Within loop | chr6:137420000-137425000 | chr6:137535000-137540000 | 115000 | GM12878 | Blood |
| rs17175127 | EAS | 0.9313 | Within loop | chr6:137420000-137425000 | chr6:137535000-137540000 | 115000 | GM12878 | Blood |
| rs17175127 | EUR | 0.95407 | Within loop | chr6:137420000-137425000 | chr6:137535000-137540000 | 115000 | GM12878 | Blood |
| rs17175127 | SAS | 0.95785 | Within loop | chr6:137420000-137425000 | chr6:137535000-137540000 | 115000 | GM12878 | Blood |
| rs6928015 | EAS | 0.87433 | Within loop | chr6:137420000-137425000 | chr6:137535000-137540000 | 115000 | GM12878 | Blood |
| rs6928015 | EUR | 0.95446 | Within loop | chr6:137420000-137425000 | chr6:137535000-137540000 | 115000 | GM12878 | Blood |
| rs6928015 | SAS | 0.83612 | Within loop | chr6:137420000-137425000 | chr6:137535000-137540000 | 115000 | GM12878 | Blood |

African: AFR, Ad Mixed American: AMR, East Asian: ASN, European: EUR, and South Asian: SAS.

**Supplemental Table 12. Internal validation of the established nomogram.**

| Ntraining : Nvalidation | Training cohort | |  | Validation cohort | |
| --- | --- | --- | --- | --- | --- |
|  | C-index (95%CI) | AUC (95%CI) |  | C-index (95%CI) | AUC (95%CI) |
| 7 : 3 | 0.656 (0.573-0.739) | 0.656 (0.572-0.739) |  | 0.637 (0.507-0.767) | 0.637 (0.505-0.770) |
| 6 : 4 | 0.640 (0.549-0.731) | 0.641 (0.549-0.733) |  | 0.610 (0.492-0.728) | 0.609 (0.491-0.728) |
| 5 : 5 | 0.619 (0.517-0.721) | 0.619 (0.517-0.721) |  | 0.658 (0.559-0.757) | 0.658 (0.558-0.757) |
| 4 : 6 | 0.616 (0.504-0.728) | 0.616 (0.503-0.729) |  | 0.664 (0.573-0.755) | 0.664 (0.572-0.756) |
| 3 : 7 | 0.651 (0.526-0.776) | 0.651 (0.524-0.777) |  | 0.636 (0.551-0.721) | 0.636 (0.550-0.722) |

AUC: area under the receiver operating characteristic curve; CI: confidence interval; N: number.
